# Baseline data and associations between urinary biomarkers of polycyclic aromatic hydrocarbons, blood pressure, hemogram, and lifestyle among wildland firefighters

**DOI:** 10.3389/fpubh.2024.1338435

**Published:** 2024-03-06

**Authors:** Bela Barros, Ana Margarida Paiva, Marta Oliveira, Sara Alves, Filipa Esteves, Adília Fernandes, Josiana Vaz, Klara Slezakova, Solange Costa, João Paulo Teixeira, Simone Morais

**Affiliations:** ^1^REQUIMTE/LAQV, Instituto Superior de Engenharia do Porto, Instituto Politécnico do Porto, Porto, Portugal; ^2^Instituto Politécnico de Bragança, UICISA: E, Unidade de Investigação em Ciências da Saúde: Enfermagem, Instituto Politécnico de Bragança Campus de Santa Apolónia, Bragança, Portugal; ^3^Environmental Health Department, National Institute of Health Dr. Ricardo Jorge, Porto, Portugal; ^4^Department of Public Health and Forensic Sciences, and Medical School, Faculty of Medicine, University of Porto, Porto, Portugal; ^5^EPIUnit – Instituto de Saúde Pública da Universidade do Porto, Porto, Portugal; ^6^CIMO, Instituto Politécnico de Bragança, Bragança, Centro de Investigação de Montanha Campus Santa Apolónia, Bragança, Portugal; ^7^SusTEC, Instituto Politécnico de Bragança, Bragança, Sustec – Associate Laboratory for Sustainability and Technology in Inland Regions – Campus Santa Apolónia, Bragança, Portugal; ^8^LEPABE-ALiCE, Departamento de Engenharia Química, Faculdade de Engenharia, Rua Dr. Roberto Frias, Porto, Portugal

**Keywords:** firefighters health, biomonitoring, biomarkers of exposure, smoking, hydroxylated polycyclic aromatic hydrocarbons, biomarkers of effect

## Abstract

**Introduction:**

Available literature has found an association between firefighting and pathologic pathways leading to cardiorespiratory diseases, which have been linked with exposure to polycyclic aromatic hydrocarbons (PAHs). PAHs are highlighted as priority pollutants by the European Human Biomonitoring Initiative in occupational and non-occupational contexts.

**Methods:**

This cross-sectional study is the first to simultaneously characterize six creatinine-adjusted PAHs metabolites (OHPAHs) in urine, blood pressure, cardiac frequency, and hemogram parameters among wildland firefighters without occupational exposure to fire emissions (> 7 days), while exploring several variables retrieved via questionnaires.

**Results:**

Overall, baseline levels for total OHPAHs levels were 2 to 23-times superior to the general population, whereas individual metabolites remained below the general population median range (except for 1-hydroxynaphthalene+1-hydroxyacenaphtene). Exposure to gaseous pollutants and/or particulate matter during work-shift was associated with a 3.5-fold increase in total OHPAHs levels. Firefighters who smoke presented 3-times higher total concentration of OHPAHs than non-smokers (*p* < 0.001); non-smoker females presented 2-fold lower total OHPAHs (*p* = 0.049) than males. 1-hydroxypyrene was below the recommended occupational biological exposure value (2.5 μg/L), and the metabolite of carcinogenic PAH (benzo(a)pyrene) was not detected. Blood pressure was above 120/80 mmHg in 71% of subjects. Firefighters from the permanent intervention team presented significantly increased systolic pressure than those who performed other functions (*p* = 0.034). Tobacco consumption was significantly associated with higher basophils (*p* = 0.01–0.02) and hematocrit (p = 0.03). No association between OHPAHs and blood pressure was found. OHPAHs concentrations were positively correlated with monocyte, basophils, large immune cells, atypical lymphocytes, and mean corpuscular volume, which were stronger among smokers. Nevertheless, inverse associations were observed between fluorene and pyrene metabolites with neutrophils and eosinophils, respectively, in non-smokers. Hemogram was negatively affected by overworking and lower physical activity.

**Conclusion:**

This study suggests possible associations between urinary PAHs metabolites and health parameters in firefighters, that should be further assessed in larger groups.

## 1 Introduction

The International Agency for Research on Cancer (IARC) has classified the occupational exposure as a firefighter as carcinogenic to humans (Group 1) (1, 2). Hazards include heat, noise, and exposure to fire emissions composed of a vast list of harmful pollutants (i.e., particulate matter (PM), polycyclic aromatic hydrocarbons (PAHs), and other volatile organic compounds (e.g., benzene), heavy metals, phthalates, perfluoroalkyl acids, organophosphorus insecticides, dioxins, flame retardants, etc.) (3, 4). Fire hazards can vary by country due to different types of burnt vegetation and materials, climate, forest area, construction materials, urbanization, and protective and preventive measures, which influence firefighters’ exposure. Research has been emphasizing the importance of PAHs exposure due to their ubiquity, inherent toxicity, and because they are among the most abundant pollutants formed during wildfires, being of particulate relevance for wildland firefighters exposome (5, 6). IARC has classified some PAHs as known, probably, and possibly carcinogenic to humans (7). Also, the U.S. Environmental Protection Agency (USEPA) has included 16 PAHs in the list of priority pollutants (8). Exposure to PAHs has been associated with respiratory (9, 10) and cardiovascular problems (11–13). Available epidemiological studies have identified oxidative stress, systemic inflammation, hypertension, and atherosclerosis as the main pathological pathways involved in cardiorespiratory toxicological effects of PAHs (9, 13, 14). Apart from exposure during fire combat, PAHs have been identified in the air of the fire stations, off-gassing from stored personal protective equipment (PPE), dirty tools, and vehicles (15–17). Jackobsen et al. (18) suggested that a higher number of diesel-fueled vehicles at Norwegian fire stations, regular live fire training, and synthetic firefighting foams have contributed to increased carcinogenic exposure among firefighters. Moreover, lifestyle habits such as diet (e.g., grilled, barbecued, and smoked meat), tobacco consumption, second-hand smoking, cooking, and traffic pollution also contribute to the total PAHs exposure burden (19–21). Once absorbed by the human body via inhalation, ingestion, or/and dermal contact, PAHs are distributed, metabolized, and mainly excreted through urine and feces (22). Biomonitoring is a useful tool to assess occupational exposure to pollutants, and urinary hydroxylated PAH metabolites (OHPAHs) are the most important biomarkers in the context of wildland firefighting (23). Moreover, the Initiative “Human Biomonitoring for Europe (HBM4EU)” has acknowledged PAHs as priority substances that need to be characterized in occupational and non-occupational biomonitoring studies of the European population (24). Therefore, to support the development of legislation and health-based guidance values for human biomonitoring, it is of utmost importance to characterize PAHs biomarkers. Besides hazardous exposure, firefighters are also under physical and mental stressors such as strenuous exercise under elevated temperatures, long work-shifts, anxiety, and sleep disturbance, which can further increase their susceptibility to disease development (25).

Associations between OHPAHs and blood cell alterations, e.g., sister chromatid exchange, have been reported for North American wildland firefighters [United States of America (USA) (26)]. DNA damage was reported in Portuguese wildland firefighters (27) and brain alterations in the South Korean firefighter’s cohort (28). Moreover, hypertension (South Korea), inflammation [United Kingdom (UK) and USA], and cardiovascular disease (CVD) development (Canada, Denmark, USA, and South Korea) have been linked with firefighting activity (29–36). Besides being important biomarkers of disease diagnosis, hematological parameters can also be used for occupational health assessment (37–39). There is currently no information about the simultaneous evaluation of these three parameters, i.e., urinary OHPAHs, blood pressure, and hematologic status in firefighters, or their possible correlations. Furthermore, since male subjects are predominant in wildland firefighters, health effects in women have been poorly characterized.

Portugal is among the European countries most affected by wildfires, with over 10,000 forest fires registered in 2022 (40, 41). Current research on Portuguese firefighters is mainly related to exposure during fire combat activities (17, 42, 43). Additionally, PM-bound PAHs have been found at fire stations at higher levels than outdoors due to inadequate layout, building materials, internal ventilation profile, parking of firefighting vehicles in closed garages with direct access to the main buildings, and storage of contaminated PPE and tools without proper cleaning procedures or air-extraction systems, which all contribute to occupational exposure at the headquarters (16, 44, 45).

Thus, this study characterizes the baseline levels (i.e., with no recent participation in firefighting activities) of OHPAHs in Portuguese wildland firefighters and provides a general assessment of their current health status (including blood pressure, cardiac frequency, and hematologic parameters) and lifestyle choices that can influence their performance and health risks. Statistical associations between the studied (bio)markers and these characteristics were explored while subgrouping by smoking status and exploring gender differences.

## 2 Materials and methods

### 2.1 Study population

**Figure 1:**
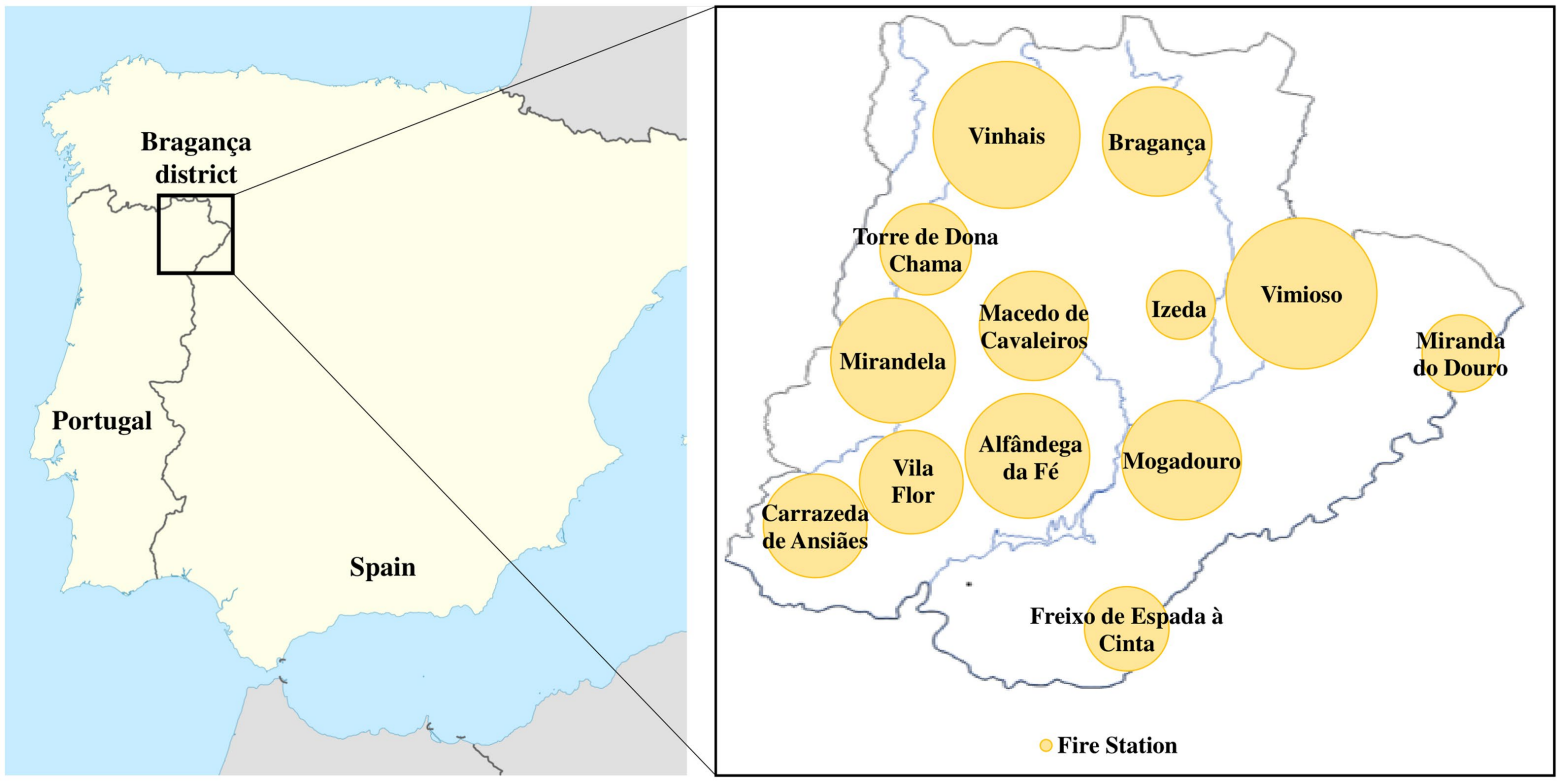
Bubble chart displaying the geographic distribution of firefighters for each enrolled fire station from the Bragança district (Northern of Portugal).

This cross-sectional study characterized firefighters from the Northern region of Portugal, one of the most affected by large and intense wildfires (additional information in Supplementary material S1.1). Firefighters from all the cities and towns of this region (i.e., Alfândega da Fé, Bragança, Carrazeda de Ansiães, Freixo de Espada à Cinta, Izeda, Macedo de Cavaleiros, Miranda do Douro, Mirandela, Mogadouro, Torre de Dona Chama, Vila Flor, Vimioso, and Vinhais) were enrolled in the study (Figure 1), which was carried out in accordance with the Declaration of Helsinki (approved protocol – Report Nr. 92/CEUP/2020 – by the Ethics Committee of the University of Porto). The subjects signed an informed consent and completed a structured questionnaire [adapted from World Health Organization (46)] self-reporting biometric characteristics (age, gender, weight, height), lifestyle (smoking, alcohol, diet within the last week, and physical activity), clinical information (medication, and existence of diagnosed diseases/health symptoms), firefighter information (years of service, career categories, signed off work leaves due to fire suppression, job tasks, mean work hours, etc.), and environmental/occupational exposure. Firefighters who acknowledged being diagnosed with neoplasia, respiratory or cardiovascular disease and having participated in firefighting activities within the last week were excluded. Given the importance of smoking on the urinary OHPAH levels, individuals were further organized into two groups: S (current smokers), and NS (non-smokers). Since there was a low participation of female firefighters, no further subdivision was made.

### 2.2 Sampling

Biological sample collection was performed during the pre-fire season (July 2021 and June 2022), i.e., during a period when firefighters had not been involved in fire combat activities for at least 7 days. Each firefighter collected a spot urine sample into a sterilized polycarbonate container. A health professional collected the blood samples following WHO guidelines (47). Transportation of collected samples was operated as requested by WHO specifications (48). Transportation and manipulation of blood samples were done on the same day of collection within 3–4 h after venipuncture, whereas urine samples were stored at −20°C until analysis.

The blood pressure measurement was executed following the regulations published by the Portuguese National Health Service (49) at the fire station in a calm and welcoming environment to ensure the correct performance of this practice. The participants were asked to be seated and relaxed for at least 5 min, to have previously emptied their bladder and refrained from smoking or ingesting stimulant drinks such as coffee in the last hour. Eating before the blood pressure measurement was not controlled.

### 2.3 Urinalysis of OHPAHs

Six urinary biomarkers of exposure to PAHs [1-hydroxynaphthalene (1-OHNaph), 1-hydroxyacenaphtene (1-OHAce), 2-hydroxyfluorene (2-OHFlu), 1-hydroxyphenanthrene (1-OHPhe), 1-hydroxypyrene (1-OHPyr), and 3-hydroxybenzo(a)pyrene (3-OHBaP)] were determined. Their extraction and quantification were performed by solid-phase extraction and high-performance liquid chromatography with fluorescence detector based on previous studies (27). The limits of detection (LODs) varied from 0.018 (1-OHPyr) to 9.59 μg/L urine (1-OHNaph+1-OHAce) whereas the respective limits of quantification (LOQs) varied from 0.06 (1-OHPyr) to 31.96 (1-OHNaph+1-OHAce) μg/L urine (50). Daily blanks and standards were analyzed to check for inter- and intra-day instrument performance. Intra-day precision was assessed through the calculation of the relative standard deviation (RSD) of triplicate urine samples injections (range: 0.1–9.6%) while inter-day precision (reproducibility) was checked every day distributed over 1 month (RSD varied 5–23%). Methodology validation yielded a recovery of 70.0–117.0% (27). Concentrations of OHPAHs were normalized with creatinine levels (μmol/mol creatinine), determined by the Jaffe colorimetric method (51).

### 2.4 Hemogram

The determined (analyzer PentraES60, Horiba Medical Diagnostics, Montpellier, France) hematological parameters were as follows: red blood count (RBC; cells ×10^6^/μL), hemoglobin (HGB; g/dL), hematocrit (HCT;%), mean corpuscular volume (MCV; fL), mean corpuscular hemoglobin (MCH; pg), mean corpuscular hemoglobin concentration (MCHC; g/dL), red cell distribution width (RDW; %); platelet count (PLT; cells ×10^3^/μL) and mean platelet volume (MPV; fL), plateletcrit (PCT; %), platelet distribution width (PDW;%); white blood count (WBC; cells ×10^3^/μL), and differentiated cells percentage (%) and count (cells ×10^3^/μL): neutrophils (NEU), lymphocytes (LYM), monocytes (MON), eosinophils (EOS), basophils (BAS), atypical lymphocytes (ALY), and large immature cells (LIC).

### 2.5 Statistical methods

The statistical software SPSS (IBM statistics 29) was used. Whenever the concentration of a OHPAH was below its LOD, the concentration was replaced by its LOD divided by √2 for statistical purposes (52). Since normal distribution was not observed by Kolmogorov–Smirnov test (*p* ≤ 0.05) for most (bio) markers, statistical differences were verified by the non-parametric Mann–Whitney U test for independent samples (or independent-samples Kruskal-Wallis Test for more than two categories). However, a normal distribution (Kolmogorov–Smirnov test, *p* ≥ 0.05) was observed for body mass index (BMI), heart beats per minute, LYM (percentage and count), MON (count), RBC, HCT, PLT, PCT, and PDW for which independent samples *t* tests were applied. Differences and possible correlations of individual and total concentration of OHPAHs (ΣOHPAHs), blood pressure and hemogram within groups (NS versus S) and across categorical variables retrieved from the self-reported questionnaire data were explored by Spearman’s rank correlation test. Statistical significance was defined as *p* ≤ 0.05 (two-tailed). For those physiological levels that have different guideline values according to gender, results were shown separately for RBC, HGB, HCT and compared accordingly with their respective reference values available for each gender. Non-parametric tests, i.e., Mann–Whitney U test, were used to avoid sample size effects. The significance of the *p*-value was also set below 0.05, which reduces the likelihood of observing significant differences due to random chance.

## 3 Results

### 3.1 Study population

The enrolled 135 firefighters (median of 36.1 years old) were mainly male (86.7%) and presented 1 to 43 years of service (Table 1). The NS group was composed of 76 subjects (56.3%, 66 males and 10 females), whereas 59 individuals (43.7%, 51 males and 8 females) were included in the S group. Firefighters smoked a mean of 15.7 ± 8.9 cigarettes per day (females tended to smoke less: median 10 cigarettes a day; *p* = 0.093), with a median smoking duration of 15.9 years (Table 1). Among NS, females presented significantly shorter firefighter careers than males, i.e., 8 versus 16.5 years of service (*p* = 0.045). The age range was similar, i.e., 20–65 years old for NS and 19–60 years old for the S group; there were no significant differences in the years of service (NS: 16.0 ± 9.9, S: 14.2 ± 10.6; *p* = 0.160) and mean BMI (NS: 27.4 ± 3.8 kg/m^2^ [females had non-significant lower BMI: 24.7 versus 28.6 (males), *p* > 0.05], S: 27.2 ± 4.4 kg/m^2^; *p* = 0.879) (Table 1). Moreover, 68.8% of firefighters were overweight (≥25 kg/m^2^). Regarding physical activity, 22.6% of firefighters acknowledged not practicing exercise, 30.5% practicing sometimes a year, 38.3% weekly, and 8.6% daily (Supplementary Table S1).

**Table 1:** Characteristics of the Portuguese firefighters.

	Non-smoker (*n* = 76)	Smoker (*n* = 59)	Total (*n* = 135)
Age (years), mean ± SD (median, min.–max.)	38.1 ± 11.3 (38.0, 20–65)	33.4 ± 10.1 (32.0, 19–60)	36.1 ± 11.0 (36.0, 19–65)
BMI (kg/m^2^), mean ± SD (median, min.–max.)	27.4 ± 3.8 (26.7, 18.5–38.7)	27.2 ± 4.4 (27.3, 18.9–41.3)	27.3 ± 4.0 (26.7, 18.5–41.3)
Female (%)	13.2	13.6	13.3
Male (%)	86.8	86.4	86.7
Number of smoked cigarettes per day, mean ± SD (median, min.–max.)	n.a.	15.7 ± 8.9 (15.0, 1–50)	n.a.
Number of years as a smoker, mean ± SD (median, min.–max.)	n.a.	15.9 ± 10.4 (13, 2–45)	n.a.
Years of service as a firefighter, mean ± SD (median, min.–max.)	16.0 ± 9.9 (14.3, 2–39)	14.2 ± 10.6 (9.0, 1–43)	15.2 ± 10.2 (12.0, 1–43)
Work demands (yes, %):			
Mental	2.7	5.2	3.8
Physics	13.5	10.3	12.1
Both	83.8	84.5	84.1
Having a health problem during the last month (yes, %):			
Cough (many times)	6.8	5.1	6.1
Phlegm (most days)	5.5	20.3	12.1
Wheezing or chest tightness while breathing	4.1	6.9	5.3

Max., maximum; Min., minimum; n.a., not applicable; SD, standard deviation.

Concerning firefighter hierarchical position and related activities, most individuals were 3rd grade firefighters (51.3%), followed by 2nd grade (18.8%), subchief (12.8%), 1st grade (10.3%), chief (4.3%), and other (2.5%). Most firefighters spend more than 8 h per day at the fire station (84.2%), performing a maximum of six different tasks (i.e., permanent intervention team (35.0%), driver (30.9%), paramedic (29.3%), administrative board, commander, rescue, diver, telephone operator, stock management, or other) (Supplementary Table S1). In this study, during their work-shift, subjects reported to be exposed to air pollution (gaseous pollutants and/or PM – 87.2%) and solvents (28.6%) on a weekly and/or daily basis (Supplementary Table S1). Few subjects lived near (~ 500 m) a factory (3.1%), industrial area (5.7%), or (~ 200 m) a farming area in which pesticides were used (27.3%) (Supplementary Table S1). Moreover, 84% of the subjects identified their activity as being both physically and mentally demanding (Table 1). Only 12.2% of subjects reported having a health problem during the last year, and 2.3% in the last 2 months. Few firefighters reported having cough, phlegm, wheezing, or chest tightness while breathing (5.1–12.1%), feeling more tired than other people of the same age (13.1%), and feeling shortness of breath while climbing a ladder (5.3%) within the last month. Also, 12.4% of the study population reported taking chronic medication (Supplementary Table S1).

### 3.2 Urinary biomarkers of exposure to PAHs

**Figure 2:**
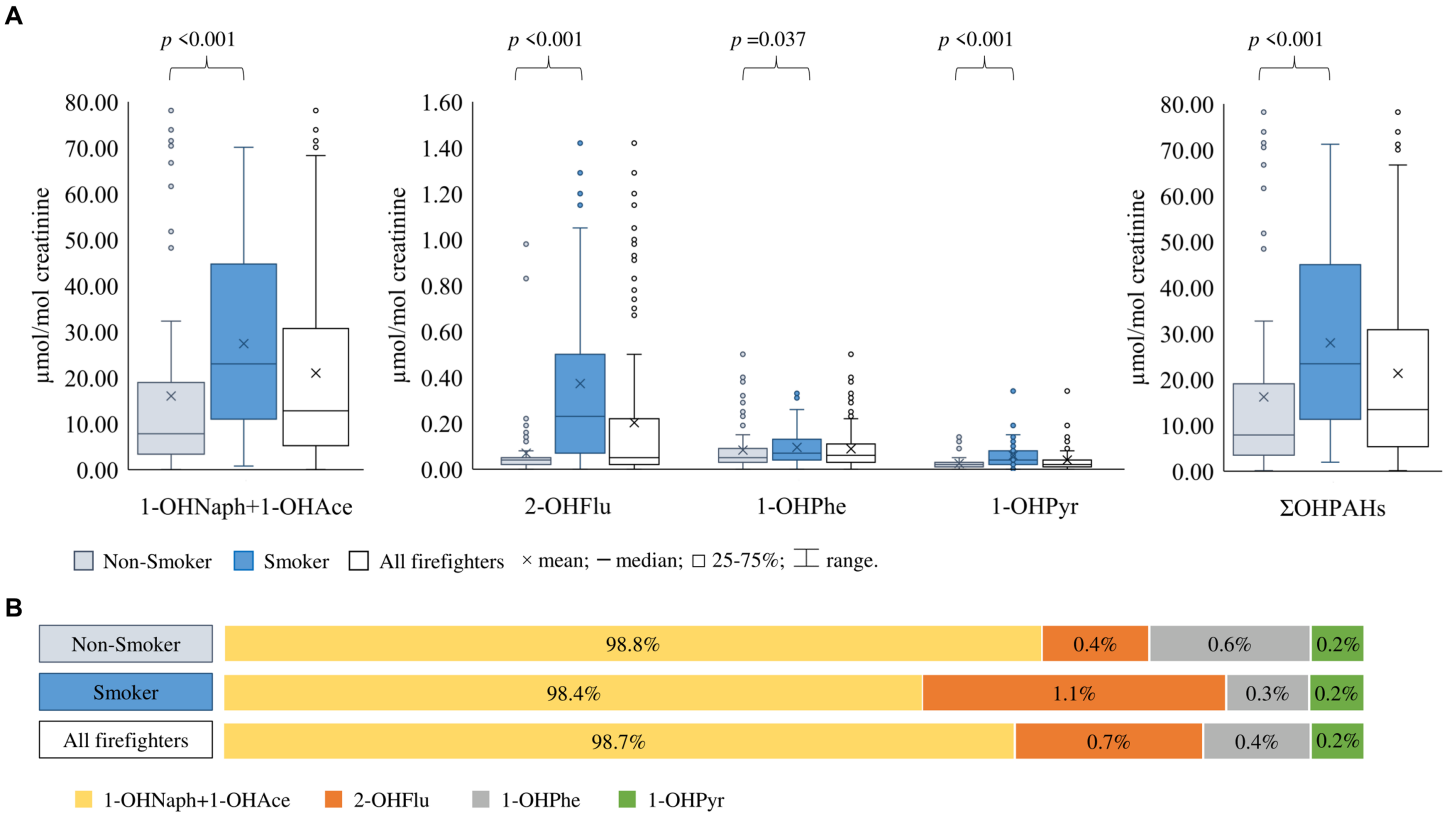
Individual and total concentrations of urinary PAH metabolites (∑OHPAHs) detected in firefighters (data expressed as µmol/mol creatinine) **(A)** and distribution (%) of the different OHPAHs **(B)** Statistical significance set at *p* < 0.05 using the Mann-Whitney U test for independent samples.

To decrease inter-individual variation due to urine dilution or differences in hydration status, creatinine adjusted levels of OHPAHs (μmol/mol creatinine) are presented in Figure 2A; unadjusted levels (μg/L) can be found in Supplementary Table S2. No differences were observed in creatinine according to smoking status (*p* = 0.175). All urinary OHPAHs were detected in 90–97% of samples, except for 3-OHBaP – metabolite of the marker of exposure to carcinogenic PAHs (7).

**Figure 3:**
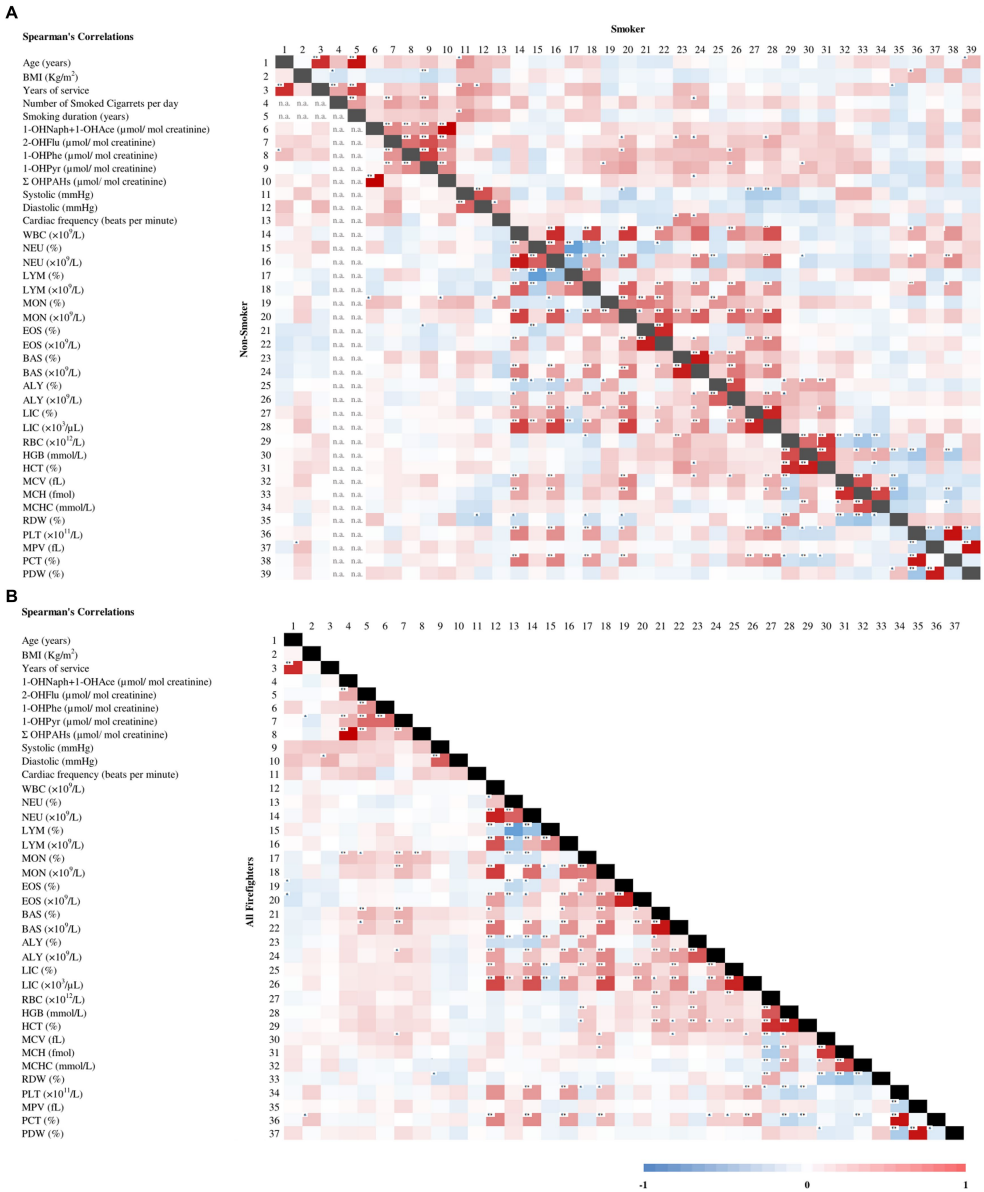
Spearman's rank correlation found between population characteristics, blood pressure, cardiac frequency, and the determined urinary and blood (bio)markers in **(A)** non-smoker and smoker individuals; and **(B)** all firefighters. 1-OHNaph+1-OHAce: 1-hydroxynaphthalene+1-hydroxyacenaphtene; 2-OHFlu: 2-hydroxyfluorene; 1-OHPhe: 1-hydroxyphenanthrene; 1-OHPyr: 1-hydrpxypyrene; ALY: Atypical lymphocytes; BAS: Basophils; EOS: Eosinophils; fL: femtoliter; HBG: Hemoglobin; LIC: Large immature cells; LYM: Lymphocytes; MCH: Mean corpuscular hemoglobin; MCV: Mean corpuscular volume; MON: Monocytes; MPV: Mean platelet volume; n.a.: Not available; NEU: Neutrophils; PCT: Plateletcrit; PLT: Platelet count; RDW: Red blood cell distribution range; WBC: White Blood Cell count. *Correlation is significant at the 0.05 level (2-tailed); **Correlation is significant at the 0.01 level (2-tailed).

ΣOHPAHs in the S group reached a median value of 23.40 μmol/mol creatinine, significantly higher (*p* < 0.001) than in the NS group (7.87 μmol/mol creatinine) (Figure 2). In this study, 45.8% of subjects smoked more than 20 tobacco cigarettes per day. Moreover, moderate to strong positive significant correlations (*r* = 0.366–0.999; *p* < 0.001–0.004) were obtained between all urinary metabolites and the ΣOHPAHs within the S group (Figure 3A); this was not observed for all OHPAHs in NS, nor when all firefighters were included (Figure 3B), NS females presented 2-fold lower urinary concentrations of ΣOHPAHs (*p* = 0.049) than NS males.

The most abundant OHPAHs (Figure 2B) were 1-OHNaph+1-OHAce (98.7%), followed by 2-OHFlu (0.7%), 1-OHPhe (0.4%), and 1-OHPyr (0.2%). Urinary PAHs’ biomarkers were 3-, 6-, and 2-fold higher in the S group than in the NS group for 1-OHNaph+1-OHAce, 2-OHFlu, 1-OHPyr, respectively (*p* < 0.001; Figure 2A). 1-OHPhe median concentration had the lowest difference in the S group compared to the NS group (40%), but it still reached statistical significance (0.07 versus 0.05 μmol/mol creatinine, *p* = 0.037; Figure 2A). Urinary 2-OHFlu (*r* = 0.379, *p* = 0.003) and 1-OHPyr (*r* = 0.359, *p* = 0.006) were significantly correlated with the number of smoked cigarettes per day (Figure 3A). Regarding gender differences, only 1-OHNaph+1-OHAce metabolite was borderline significantly lower in females in comparison to males (4.16 versus 8.57 μmol/mol creatinine; *p* = 0.053) within the NS group; no significant differences were observed for the other exposure biomarkers. Urinary 1-OHPyr was inversely correlated with BMI in all firefighters (*r* = −0.187; *p* = 0.030), especially in the S group (*r* = −0.336; *p* = 0.009). Interestingly, BMI was also negatively correlated with the number of cigarettes smoked per day (*r* = −0.216; *p* = 0.042).

The ΣOHPAHs was 3.5-fold higher in firefighters who acknowledged exposure to gaseous pollutants and/or PM during their work-shift in comparison to the ones who did not (*p* = 0.006; Supplementary Table S3). No differences in individual or ΣOHPAHs were found between subjects who indicated additional exposure to smoke in the 5–10 km surrounding their workplace and those who did not. However, for subjects who reported additional exposure within 5–10 km, differences in urinary 1-OHPyr concentrations were observed among firefighters from different fire stations (*p* = 0.034; Supplementary Figure S1). Subjects from the Bragança fire station presented 5-times higher median 1-OHPyr levels than those from Carrazeda de Ansiães (Supplementary Figure S1). However, based on the information gathered from the questionnaires, no significant associations were found (data not shown) with any of the variables representing potential sources of exposure (type of diet, heating use, candle lighting, and pesticide use).

### 3.3 Blood parameters

Blood pressure, cardiac frequency, hemogram parameters, and their respective reference values (considered normal for the Portuguese population) are displayed in Table 2.

**Table 2:** Blood pressure, cardiac frequency, and hemogram characteristics of the studied Portuguese firefighters [data presented as median (minimum-maximum)] and *p* value of statistical tests for distribution differences between non-smoker and smoker firefighters (Independent-samples Mann–Whitney U test, unless indicated otherwise).

	Non-smoker	Smoker	*p* value	Total	Reference range
**Blood pressure**					(53)
Systolic (mmHg)	129.5 (114–170)	135 (109–209)	0.09	134 (109–209)	120
Diastolic (mmHg)	85 (69–113)	85 (70–128)	0.63	85 (69–128)	80
**Cardiac frequency**					
Beats per minute	69.5 (49–113)	73 (53–100)	0.61*	71 (49–113)	60–100
**Hemogram**					**⸶** (54);**⸹** (55); ¥ (56); §: (57)
WBC (×10^9^/L)	7.20 (4.40–15.50)	7.20 (4.40–12.70)	0.59	7.20 (4.40–15.50)	4.5–11.0 × 10^9^/L ⸶
NEU (%)	55.40 (32.80–70.60)	54.60 (29.70–68.00)	0.42	54.90 (29.70–70.60)	54–62% ⸶
NEU (×10^9^/L)	3.65 (1.97–10.98)	3.82 (1.69–7.82)	0.69	3.78 (1.69–10.98)	1.5–8.0 × 10^9^/L ⸹
LYM (%)	33.10 (20.40–54.40)	33.20 (19.30–56.80)	0.51*	33.20 (19.30–56.80)	25–33% ⸶
LYM (×10^9^/L)	2.32 (1.30–4.13)	2.50 (1.33–5.22)	0.21*	2.43 (1.30–5.22)	0.8–4.0 × 10^9^/L ⸹
MON (%)	7.45 (4.00–13.90)	8.00 (5.40–11.10)	0.19	7.60 (4.00–13.90)	3–7% ⸶
MON (×10^9^/L)	0.54 (0.32–1.10)	0.59 (0.33–1.00)	0.12*	0.55 (0.32–1.10)	≤1.2 × 10^9^/L ⸹
EOS (%)	2.20 (0.70–9.70)	2.30 (1.00–7.10)	0.58	2.20 (0.70–9.70)	1–3% ⸶
EOS (×10^9^/L)	0.16 (0.05–0.72)	0.17 (0.07–0.49)	0.52	0.17 (0.05–0.72)	≤0.3 × 10^9^/L ⸹
BAS (%)	0.70 (0.30–3.10)	0.90 (0.40–10.60)	0.01	0.80 (0.30–10.60)	≤0.75% ⸶
BAS (×10^9^/L)	0.05 (0.01–0.19)	0.07 (0.02–1.24)	0.02	0.05 (0.01–1.24)	≤0.3 × 10^9^/L ⸹
ALY (%)	0.90 (0.07–3.40)	1.00 (0.50–2.50)	0.11	0.90 (0.07–3.40)	n.a.
ALY (×10^9^/L)	0.07 (0.03–0.80)	0.07 (0.04–0.25)	0.22	0.07 (0.03–0.80)	n.a.
LIC (%)	0.70 (0.30–2.60)	0.80 (0.30–1.40)	0.42	0.80 (0.30–2.60)	n.a.
LIC (×10^3^/μL)	0.05 (0.02–0.20)	0.05 (0.01–0.18)	0.46	0.05 (0.01–0.20)	1.0 × 10^3^/μL §
RBC (×10^12^/L)	5.03 (4.07–6.05)	5.20 (4.26–6.37)	0.05*	5.06 (4.07–6.37)	
Male	5.01 (4.07–6.05)	5.16 (4.26–5.98)	0.17*	5.05 (4.07–6.05)	4.3–5.9 × 10^12^/L ⸶
Female	5.06 (4.14–5.46)	5.39 (4.77–6.37)	0.08*	5.11 (4.14–6.37)	3.5–5.5 × 10^12^/L ⸶
HGB (mmol/L) ^a^	9.74 (7.76–1.92)	9.99 (8.19–12.85)	0.07	9.87 (7.76–12.85)	
Male	9.62 (7.76–11.92)	9.99 (8.19–10.92)	0.09	9.81 (7.76–11.92)	8.38–10.86 mmol/L ⸶
Female	9.90 (7.88–10.74)	10.18 (9.12–12.85)	0.36	9.93 (7.88–12.85)	7.45–9.93 mmol/L ⸶
HCT (%)	46.85 (37.00–54.70)	48.10 (39.00–61.80)	0.03*	47.50 (37.00–61.80)	
Male	46.75 (37.00–54.70)	47.95 (39.00–54.10)	0.09*	47.45 (37.00–54.70)	41–53% ⸶
Female	47.25 (38.10–49.30)	48.70 (44.10–61.80)	0.12*	47.60 (38.10–61.80)	36–46% ⸶
MCV (fL)	93.00 (73.00–103.00)	93.00 (75.00–100.00)	0.70	93.00 (73.00–103.00)	80–100 fL ⸶
MCH (fmol) ^b^	1.94 (1.43–2.20)	1.92 (1.43–2.14)	0.61	1.93 (1.43–2.20)	1.55–2.17 fmol ⸶
MCHC (mmol/L) ^a^	20.98 (19.05–21.97)	20.98 (18.99–21.53)	0.89	20.98 (18.99–21.97)	19.25–22.36 mmol/L ⸶
RDW (%)	10.90 (9.80–5.60)	10.90 (9.70–13.10)	0.38	10.90 (9.70–15.60)	11.5–14.5% ⸶
PLT (×10^11^/L)	2.23 (0.54–3.46)	2.30 (1.13–3.56)	0.30*	2.24 (0.54–3.56)	1.5–4.0 × 10^11^/L ⸶
MPV (fL)	9.10 (7.70–11.30)	9.10 (7.70–11.60)	0.45	9.10 (7.70–11.60)	6.5–12.4 fL ⸹
PCT (%)	0.20 (0.05–0.31)	0.21 (0.10–0.30)	0.44*	0.21 (0.05–0.31)	0.10–0.50% ¥
PDW (%)	16.15 (11.00–21.30)	16.00 (11.80–22.50)	0.39*	16.00 (11.00–22.50)	10.0–18.0% ¥

* *p* value obtained by *t*-test for Equality of Means (significance < 0.05); ¥, these recommended values are set for Brazilian Portuguese general population; %, percentage; ALY, atypical lymphocytes; BAS, basophils; EOS, eosinophils; fL, femtoliter; HBG, hemoglobin; HCT, hematocrit; LIC, large immature cells; LYM, lymphocytes; MCH, mean corpuscular hemoglobin; MCHC, mean corpuscular hemoglobin concentration; MCV, mean corpuscular volume; MON, monocytes; MPV, mean platelet volume; n.a., not available; NEU, neutrophils; PCT, plateletcrit; PDW, platelet distribution width; PLT, platelet count; RBC, red blood cell count; RDW, red blood cell distribution range; WBC, white blood cell count. *p* value is a result from Independent-Samples Mann–Whitney U Test (significance < 0.05, in bold).

a Data were converted from g/dL to mmol/L.

b Data were converted from pg to fmol.

**Figure 4:**
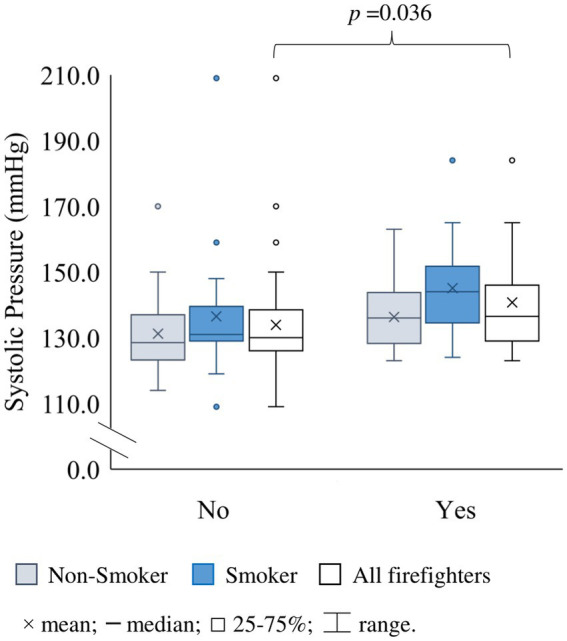
Systolic pressure distribution in Portuguese firefighters based on their inclusion in the permanent intervention team (No *versus* Yes) (data expressed as mmHg). Statistical significance set at *p* < 0.05 using the Mann-Whitney U test for independent samples.

Overall, median diastolic and systolic blood pressure were 134 and 85 mmHg, respectively. No differences were observed by gender. Blood pressure in firefighters exceeded the 120 and 80 mmHg guidelines for optimal blood pressure set by the Portuguese Hypertension Society (53). Among all subjects, 28% could be considered hypertensive. However, 71% of firefighters presented values higher than the considered optimal blood pressure; of these, 39% showed values corresponding to hypertension (≥140/90 mmHg). On the other hand, the median heartbeat was 71 beats/min for all firefighters, and only 18% presented a cardiac frequency below the normal range (60–100 heartbeats/min) (58); only 3% exceeded 100 beats/min (Table 2); no differences by gender (*p* > 0.05). No significant differences were found in systolic or diastolic pressure, and cardiac frequency between S and NS firefighters (*p ≥* 0.09; Table 2). Still, there was an almost 2-fold higher frequency of individuals with hypertensive measures (≥140/90 mmHg) in the S than in the NS group (36% versus 19%; Table 2). A significant positive correlation between systolic pressure and duration of smoking (in years) was found (*r* = 0.419; *p* = 0.017; Figure 3A), Additional significant positive correlations were found within the S group (Figure 3A), i.e., (i) age and systolic pressure (*r* = 0.416; *p* = 0.016); and (ii) both systolic and diastolic pressure with years of service (*r* = 0.426, *p* = 0.013; and *r* = 0.357; *p* = 0.041, respectively). Firefighters who were part of the permanent intervention team showed a 5% increase in systolic pressure in comparison to those who were not (*p* = 0.036; Figure 4), no significant differences were found for other variables retrieved from the questionnaires (*p* > 0.05).

Regarding hemogram parameters, the measured WBCs were within the normal range for the Portuguese population (55) for both NS and S groups (Table 2). However, the median percentage for the different leucocyte types was predominantly near (LYM) or slightly above the upper normal limit (MON and BAS), principally for smokers. An inversion was observed for the percentage of NEU since median levels were near the minimum normal percentage (Table 2). Female NS firefighters presented higher number of LYM (+30.6%, *p* = 0.005) and lower MON percentage (−5.3%, *p* = 0.034) at baseline. Women firefighters who smoke presented higher ALY (+28.6%, *p* = 0.037) than men. Even so, the median ALY percentage in the Portuguese firefighters were normal (0.90%; Table 2), and 61% of firefighters presented optimal ALY values. As for LIC, median percentage was below the recommended level (1%), but 12% of firefighters presented LIC above this percentage (LIC maximum of 2.60%). Nevertheless, the median number of LIC [0.05 (0.01–0.20) × 10^3^/μL] was below the reference values, i.e., 1.0 × 10^3^/μL (57). No other differences by gender were observed.

**Figure 5:**
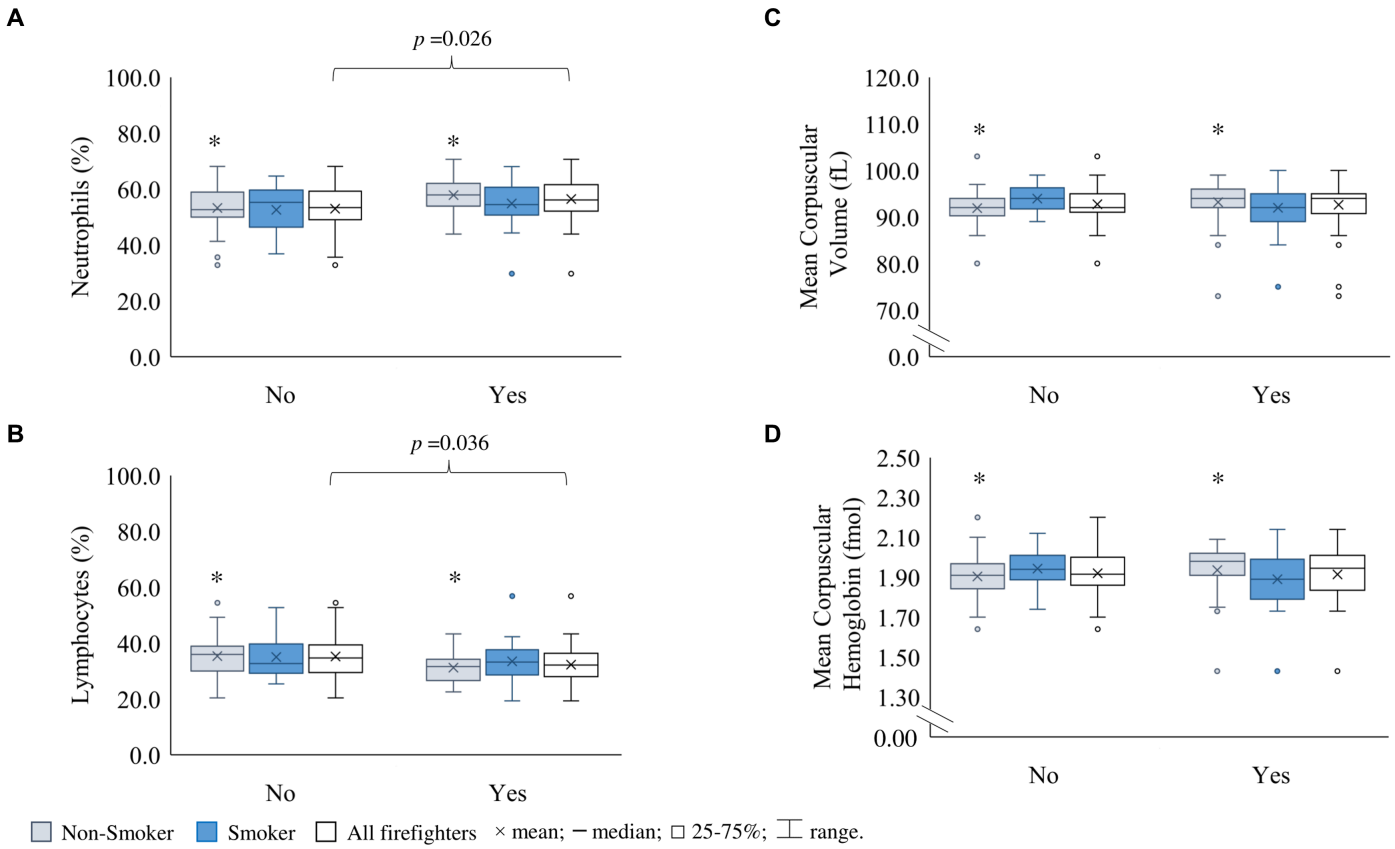
Hemogram parameters among firefighters who have acknowledged exposure to smoke/air pollutants in a 5-10 km radius from their workplace (No *versus* Yes). **(A)** Neutrophils (%); **(B)** Lymphocytes (%); **(C)** Mean corpuscular volume (fL); **(D)** Mean corpuscular hemoglobin (fmol). *Significant differences between "No" and "Yes" within the non-smoker group for **(a)** *p* = 0.011; **(b)** *p* = 0.017; **(c)** *p* = 0.013; **(d)** *p* = 0.035. Statistical significance set at *p* < 0.05 using the Mann-Whitney U test for independent samples.

The maximum LYM concentration of 5.22 × 10^9^/L in the S group was above the set reference limit (Table 2). Moreover, besides smokers presenting a significantly higher BAS than non-smokers (28% higher BAS percentage, *p* = 0.01, and 40% BAS count, *p* = 0.02; Table 2), there was also a significant correlation between BAS count and the number of smoked cigarettes per day (*r* = 0.281; *p* = 0.044; Figure 3A). Although non-significant, there were also higher median leucocyte type counts in the S group in comparison to the NS, i.e., 3.82 × 10^9^/L versus 3.65 × 10^9^/L for NEU, 2.50 × 10^9^/L versus 2.32 × 10^9^/L for LYM, and 0.59 × 10^9^/L versus 5.40 × 10^8^/L for MON, respectively (Table 2). Firefighters who reported having exposure to smoke (within 5–10 km radius from their workplace) presented significantly higher NEU (+5.2%; *p* = 0.026) and lower LYM (−7.2%; *p* = 0.036) percentage than those who did not (Figures 5A,B). These associations were stronger among non-smokers (NEU: +10.1%, *p* = 0.011; LYM: −12.1%, *p* = 0.017; Figures 5A,B). A 16% reduction in LYM count was observed in firefighters who practice physical activity weekly versus sometimes/year (*p* = 0.006; Supplementary Table S3). Moreover, spending more than 10 h a day at the fire station decreased NEU (7%, *p* = 0.038) and increased LYM (11%, *p* = 0.022), when compared to spending 8–9 h (Supplementary Table S3). Lastly, subjects who were drivers had a significantly decreased percentage of MON (7%, *p* = 0.032) and ALY (10% *p* = 0.021) (Supplementary Table S3).

Median levels were within the normal range for RBC, HGB, HCT, MCV, MCH, MCHC, RDW, PLT, MPV, PCT, and PDW (Table 2); no significant differences by gender were observed. Despite being within normal, irrespectively of gender, HCT percentage was significantly higher in the S group than in the NS group (48.10% versus 46.85%, *p* = 0.03) while RBC and HBG were borderline non-significant among these subgroups (*p* = 0.05 and *p* = 0.07, respectively; Table 2). A significantly negative correlation between RDW and years of service (*r* = −0.313, *p* = 0.025; Figure 3A) was found within the S group.

Work environment characteristics such as exposure to smoke, PM, and/or gaseous pollutants during firefighters’ work-shifts were associated with a slight decrease in MCHC (1%; *p* = 0.011; Supplementary Table S3). Moreover, reporting solvent exposure during the work-shift was associated with a decreased HGB (4%; *p* = 0.009) and increased PCT (10%; *p* = 0.027) (Supplementary Table S3). Being a driver was associated with a 7% decrease in HGB, a 5% reduction in HCT, and a 11% increase in PCT (*p* ≤ 0.021; Supplementary Table S3). Significant differences in MCV (fL) and MCH (fmol) were only observed in NS firefighters who reported having exposure to smoke/air pollutants within a 5–10 km radius from work in comparison to those who were not exposed (MCV: +2.2%, *p* = 0.013; MCH: +3.7%, *p* = 0.035; Figures 5C,D). Regarding lifestyle characteristics, firefighters who exercise weekly presented significantly lower PLT count (−13%, *p* = 0.005) and PCT (−12%, *p* = 0.006) than those who exercise only sometimes a year, while the latter presented significantly reduced PDW (−8%, *p* = 0.005) and higher PLT count (+15%, *p* = 0.019) than firefighters who do not exercise (Supplementary Table S3). Additionally, BMI was positively correlated with PLT count in the S group (*r* = 0.291, *p* = 0.034; Figure 3A), MPV in the NS group (*r* = 0.245, *p* = 0.044; Figure 3A), and PCT in all firefighters (*r* = 0.213, *p* = 0.009; Figure 3B).

### 3.4 Correlations between biomarkers of exposure and health (bio)markers

No associations were found between PAH metabolites (individual or ∑OHPAHs) with blood pressure parameters (Figure 3). Considering hemogram parameters, a positive correlation between 1-OHNaph+1-OHAce and MON was found in non-smokers (Figure 3A) and all firefighters (Figure 3B), and because these urinary metabolites were the main contributors to ΣOHPAHs, the latter was also correlated with MON (Figure 3). For the other PAH metabolites, moderate positive correlations were found for all firefighters: (i) 2-OHFlu with MON and BAS (0.199 < *r <* 0.297; *p* ≤ 0.029) and (ii) 1-OHPyr with MON, BAS, ALY, and MCV (0.188 < r < 0.309; *p* ≤ 0.039) (Figure 3B). These correlations were positive and predominantly significant in the S group (0.283 < *r <* 0.384; *p* ≤ 0.042 except for 1-OHNaph+1-OHAce and ΣOHPAHs, *p* > 0.05; Figure 3A). 1-OHPhe was correlated with MON (*r* = 0.341; *p* = 0.013) and ALY (*r* = 0.310; *p* = 0.024) for smokers only (Figure 3A). However, 2-OHFlu, 1-OHPhe, and 1-OHPyr were not correlated with any immunologic parameter in the NS group. On the other hand, an inverse correlation was observed for NEU and EOS and 2-OHFlu and 1-OHPyr, respectively, only in the NS group.

Interestingly, there were negative correlations between systolic pressure and MON and LIC in the S group, and systolic pressure with RDW without subgrouping (Figure 3), whereas diastolic pressure was negatively correlated with RDW in the NS group. Regarding cardiac frequency, the results displayed a positive correlation with MON and BAS in the NS and S groups, respectively (Figure 3A).

## 4 Discussion

### 4.1 Study population

This study characterizes firefighters contributing for a baseline profile of these workers without any exposure to fire combat activities. The frequency of smokers in Portuguese firefighters (47.3%) is comparable with the ones reported for USA firefighters in the last decades, i.e., 41.6–51.3% of smokers (59–61). The frequency of overweight firefighters (68.8%) is higher than what was observed for the general Portuguese population (55.9%) and the northern Portuguese population (58.6%) in 2019 (62). Available literature suggests an association between weight gain and working as a firefighter, specifically an increase of 0.5–1.5 kg per year (63, 64). Accordingly, 10 years of service could represent a weight augment of 5 to 15 kg. In fact, more than 60% of our study population had more than 10 years of service, which could help explain the high frequency of overweight individuals. However, these data should be considered with caution since recent studies have highlighted that the percentage of body fat is a better measurement of obesity than BMI (65, 66) mainly because weight can reflect a higher muscle mass rather than body adipocyte mass, thus leading to possible misclassification of obesity. Moreover, the self-reported low physical activity in most of the subjects (Table 1) is concerning because sedentarism has also been linked to higher cardiovascular risk among firefighters (11, 67).

### 4.2 Urinary biomarkers of exposure to PAHs

This study characterizes firefighters who did not participate in fire combat activities within the last week. This period was selected based on urinary excretion half-lives of 6–35 h (1-OHPyr via inhalation) and 2.3–23.5 h (1-OHNaph, 2-OHFlu, 1-OHPhe, and 1-OHPyr via ingestion), and 13 h (1-OHPyr via dermal contact) (3, 68, 69) yet inhalation and dermal route data is still limited to 1-OHPyr, and information regarding excretion rates based on all routes of exposure and possible chemical interactions in mixed exposures is lacking. The non-detection of 3-OHBaP has been previously observed since it is mainly excreted through feces (70). The median ΣOHPAHs is 2 to 14-times higher than the range of mean/median concentrations that have been reported for firefighters not exposed to fire emissions [0.95–6.96 μmol/mol creatinine; (69)]. For smokers, there is a known contribution from tobacco consumption to urinary OHPAHs (71–73). The significantly higher levels in smokers (Figure 2) could be related to the number and type of cigarettes smoked per day, since significant correlations between OHPAH and number of smoked cigarettes a day were also found, highlighting a common and prevalent route of exposure to PAHs in the S group. Regardless of smoking status, firefighters presented 5–8 times higher median creatinine adjusted ΣOHPAHs than non-occupationally exposed Italian population (74–77), whereas non-adjusted median values (μg/L) were 11 to 23-times higher than those for the Slovenian and Italian population (74, 78). The identified sources of exposure in these studies were diet, biomass-burning emissions from heating systems during winter, living near busy roads or near a waste-to-energy incinerator (74, 78). On the other hand, in the present study, subjects did not use biomass-burning heating systems during sample collection, nor was the fire station located near incineration centers, suggesting other potential sources of PAHs exposure for this population. As for other countries, Portuguese firefighters displayed ΣOHPAHs concentrations that were 2-fold above those reported by the USA National Health and Nutrition Examination Survey (NHANES: 2009–2016) (79), and 2-times below the ΣOHPAHs reported for the urban Wuhan-Zhuhai cohort in China (80). The latter populations were from heavily polluted cities (81), which helps explaining the high metabolite concentrations found in Chinese.

The sequence of most abundant OHPAHs (Figure 2B) is similar to what has been previously described in Portuguese and USA firefighters (27, 82–85). The significant difference found in individual OHPAHs according to smoking status (Figure 2) reflect the impact of smoking on urinary levels of these compounds. Moreover, the unequally significant distribution of 2-OHFlu concentrations between the NS and S groups (Figure 2B) suggests that tobacco smoke is a main source of fluorene exposure in the characterized Portuguese firefighters. The correlations found between 2-OHFlu and 1-OHPyr with smoked cigarettes per day, support the hypothesis that these two compounds are among the most selective biomarkers of tobacco consumption. These results are in accordance with Helen et al. (72), who also suggested that hydroxyfluorenes and 1-OHPyr may have discriminant power regarding smokers and non-smokers. Since no differences were observed in creatinine levels by smoking status, an influence of PAHs exposure in creatinine levels in association with cigarette smoking was not observed. Nevertheless, ongoing research has conflicting results concerning the influence on estimated glomerular filtration rate in smokers (86), thus further studies should aim to understand the impact of PAHs exposure in glomerular filtration rate and if impacts urinary creatinine clearance. In comparison to the general population, individual 2-OHFlu, 1-OHPhe, and 1-OHPyr (μmol/mol creatinine) in the Portuguese population [2-OHFlu: 0.13–0.16; 1-OHPhe: 0.06–0.08; 1-OHPyr: 0.04–0.06; (87)], the USA NHANES [2-OHFlu: 0.16; 1-OHPhe: 0.07; 1-OHPyr: 0.07; (79)], Italy [2-OHFlu: 0.07–0.11; 1-OHPhe: 0.05–0.10; 1-OHPyr: 0.02–0.04; (74–77)], and Germany [1-OHPyr: median range 0.16–0.38; (88)] were higher than those determined in the characterized Portuguese firefighters (median for all firefighters: 2-OHFlu: 0.05; 1-OHPhe: 0.06; 1-OHPyr: 0.02; Figure 2A).

1-OHPyr, considered the biomarker of exposure to PAHs, has an occupational biological exposure index of 2.5 μg/L proposed by the American Conference of Governmental Industrial Hygienists (89) and the guidance level of 1.0 μmol/mol creatinine for coke oven workers with a pyrene/benzo(a)pyrene ratio equal to 2.5 (90) which, adjusted for the studied firefighters, would be equivalent to approximately 0.59 μmol/mol creatinine. These values were not surpassed in this study (maximum of 0.35 μmol/mol creatinine and 1.63 μg/L; Figure 2A; Supplementary Table S2, respectively), suggesting that most firefighters should not experience any adverse health effects from pyrene/PAHs-related exposure. Moreover, median levels for 1-OHPyr (0.02 μmol/mol creatinine) were below those reported for firefighters from Denmark, German Sweden, and USA, at baseline level before exposure to fire emissions, i.e., 0.03 to 0.72 μmol/mol creatinine (69, 88). The inverse association between 1-OHPyr and BMI (Figure 3) was also observed in other studies due to the potential effects of nicotine on appetite reduction and/or augmented metabolic rate among smokers (91–95).

Multiple sources of PAHs have been identified at fire stations including, vehicle exhaust in unventilated garages, dust, and off-gassing compounds from contaminated PPE and tools (44, 45, 83, 96). A recent UK survey (>10,000 subjects) has revealed that only one third of firefighters cleaned their PPE after use, and both cleaned and contaminated gear were often stored together (97). Even though cleaning and storage practices were not included in this study’s questionnaire, they are important to characterize possible sources of (air and surface) PAHs contamination at fire stations. On the other hand, a study conducted in 2014 (during the pre-fire season) has identified traffic emissions, lubricant oil use, and both fuel and biomass combustions as main sources of PAHs at Portuguese fire stations, which promoted lung cancer risks that exceeded 9 to 44-times the WHO-based guideline (98). For chronic PAHs inhalation, minimal risks levels for humans have only been reported for naphthalene [0.0007 ppm; (99)] by the Agency for Toxic Substances and Disease Registry and, based on studies on coke-oven workers, WHO has estimated the unit risk of genotoxic carcinogenicity for benzo(a)pyrene (has an indicator of ambient air PAHs) to be 8.7 × 10^−5^ per ng/m^3^. Available European legislation established 1 ng/m^3^ benzo(a)pyrene as the annual mean target value for human health protection (100). A previous study by Oliveira et al. (83) has quantified PM-bound PAHs at Portuguese fire stations from the same district. The reported values ranged from 46.4 to 428 ng/m^3^ for total PM-bound PAHs in the personal breathing air zone of Portuguese firefighters, which air concentrations correlated with the sum of urinary PAHs’ metabolites (*r* = 0.367–0.886). Occupational limits for total PAHs in the air has been set at 100 and 200 μg/m^3^ by the National Institute for Occupational Safety and Health (101) and the North American Occupational Safety and Health Administration, respectively (102), the median levels in the personal air of Portuguese firefighters did not surpass both limits (83). In the present study, the main contributors to ΣOHPAHs were 1-OHNaph+1-OHAce, which parent compounds have been identified as the most abundant in indoor air at Portuguese fire stations (98). Moreover, at Polish fire stations, the highest naphthalene and acenaphthene air concentrations were found in the garage and changing rooms areas (103). Indeed, the combustion of diesel and gasoline, and the evaporation/sublimation of, crude oil, petroleum products, pest repellent, deodorant, and air fresheners have been identified as sources of naphthalene (104). Therefore, even without involvement in firefighting activities over the last week, firefighters’ exposures to air pollution and solvents during their work-shift on a weekly and/or daily basis (Supplementary Table S1) could have influenced the levels of urinary OHPAHs concentrations. The observation of 3.5-fold higher ΣOHPAHs in firefighters who acknowledge exposure to PM and/or gaseous pollutants during their work-shift (Supplementary Table S3) agrees with previous studies that reported fire station contamination and firefighters’ exposure to PAHs at their headquarters (without fire combat) in Australia, Portugal, and the USA (44, 83, 105). Therefore, suggesting that there must be other significant sources of exposure to PAHs at Portuguese fire stations. There is only a single ground air monitoring station within the Bragança district (Portugal) that quantifies outdoor benzo(a)pyrene, which data were not available during the campaign days (100). No difference in individual or ΣOHPAHs was observed in firefighters with and without additional exposure to smoke in 5–10 km from their workplace. However, Bragança firefighters had 5-times higher median 1-OHPyr levels than those from Carrazeda de Ansiães (Supplementary Figure S1). Despite the Portuguese inland districts such as Bragança having less intense traffic than those on the coastline (106, 107), the city of Bragança has a higher population density and far more trafficked roads (i.e., inside 2 km radius from the fire station there are four national roads and one main itinerary) than Carrazeda de Ansiães. Indeed, some studies have identified an increase in PM_10_ and PM_2.5_ air concentrations in Bragança due to traffic pollution (108, 109).

Other sources of PAHs, e.g., diet (grilled/barbecued food, smoked meat), open burning, candle lighting, and house insecticides use (19, 21, 23) can influence the levels of biomarkers of exposure. Despite Portuguese people having a Mediterranean diet (110), the Northern region is known for increased smoked meat production and consumption. No differences were observed for OHPAHs according to diet choices, heating use, candle lighting, or pesticide use. Although cooking activities were not evaluated in this survey, a study has shown a 9-fold increment in ΣOHPAHs measured in Portuguese workers who performed grilling activities for 4.6 ± 2.2 h in restaurants compared to non-exposed workers (0.2–42.3 versus 0.097–1.66 μmol/mol creatinine), suggesting that it might also be a possible source of PAHs exposure (111).

### 4.3 Blood parameters

The European Society of Cardiology and the European Society of Hypertension have classified hypertension as a blood pressure measure at the physician’s office that is above 140/90 mmHg (53). The frequency of hypertensive firefighters (28%) was lower than the 35–40% prevalence found for the Portuguese population (112), yet, within those with higher blood pressure (> 120/80), 39% presented a pressure ≥ 140/90 mmHg. It is important to notice that having one-time measured blood pressure ≥ 140/90 mmHg is not indicative of a hypertension diagnosis, but suggests an increased risk of hypertension, thus representing an important risk factor for cardiovascular disease development in the studied population. Available literature has described a higher prevalence of hypertension among males (113). No differences were observed by gender in firefighters; this observation could be due to the age of female firefighters (median 39 years old), which could indicate a higher number of female subjects in their late 30s. At this age, hypertensive disorders related to pregnancy and female hormonal alterations (e.g., pre-menopausal state) that interact with the renin-angiotensin-aldosterone system can be associated with higher blood pressure (113). An augmented proportion of individuals with high blood pressure has also been reported among firefighters worldwide (USA, Canada, France, and South Africa), i.e., frequency of 11–69% (31, 114–116).

Smoking has been recognized as a risk factor for cardiovascular disease (53, 117, 118). However, no differences were observed between NS and S yet, the latter group presented a higher frequency of hypertensive subjects. Also, a significant correlation between blood pressure and years as a smoker was found, which has also been previously observed for non-occupational populations (118, 119). Moreover, moderate significant correlations were found within the S group, i.e., systolic pressure with age, and both systolic and diastolic pressure with years of service (Figure 3A). Therefore, these results are suggestive of an association between age, smoking duration, years of service, and blood pressure, which could cumulatively contribute to an increased risk for hypertension development in smoking firefighters.

The activities of a firefighter are predominantly stressful and can influence their physiological parameters (120, 121). The intervention team replies to any emergency call, e.g., accidents, fires, or disaster occurrences. It is possible that the permanent team members could experience higher stress levels due to the unpredictability of their workday, which could explain the higher systolic pressure found for firefighters from this team (Figure 4), yet more studies are needed to characterize this relationship.

The determined number of white blood cells (Table 2) is comparable with previously published data for UK firefighters and fire service instructors before exposure to fire combat training, i.e., 7.20 × 10^9^/L versus 5.7–7.1 × 10^9^/L for WBC, 3.78 × 10^9^/L versus 3.1–4.1 × 10^9^/L for NEU, 0.55 × 10^9^/L versus 5.0–6.0 × 10^8^/L for MON, 0.17 × 10^9^/L versus 1.0–2.0 × 10^8^/L for EOS, and 0.05 × 10^9^/L versus 3.0–8.0 × 10^7^/L for BAS, respectively (34, 122). Concerning LYM, the number of cells was slightly higher in this study than in the range found in other UK studies for firefighters and fire service instructors [2.43 × 10^9^/L versus 1.7–2.1 × 10^9^/L; (34, 122)]. However, these values remain in the normal range established for LYM count [<5.0 × 10^9^/L; (123)]. Interestingly, Watt et al. (122) reported leucocyte counts for control subjects with no heat exposure that were below those found in fire service instructors both pre- and post-exposure, suggesting a more active immune system in workers in comparison to controls, both at the baseline and after fire instruction drills. NS female firefighters had higher number LYM and lower MON percentage than NS male. García-Dabrio et al. (124) also observed higher LYM (CD3+ and CD4+) in Spanish healthy women in comparison to males while Varghese et al. (125) reported higher MON activity/recruitment in males in comparison to females. Smoking tobacco has been reported to be associated with elevated counts of NEU, LYM, MON, and/or BAS (126–130). The significantly higher BAS in S in comparison to NS firefighters support the association between smoking habits and mild basophilia among the studied population. Augmented ALY is an indicator of recent immune activity (usually towards a viral infection); values below 12% are normal, whereas optimal ones are below 1% (131). Since in this study women generally smoked less than men, the significance of having higher ALY among female smokers in comparison to males needs further study. Similarly, an increase in LIC is suggestive of recent development of viral infections and/or inflammation; in normal conditions, there are less than 1% of LIC in the whole blood (132, 133); only 12% of firefighters surpassed this percentage, yet median LIC number was within its recommended level (Table 2).

On the other hand, decreased indoor air quality due to higher air PM concentration has been highlighted as a potential factor for increased systemic inflammation (134). The observation of higher NEU and lower LYM among those who acknowledged having exposure to smoke (5–10 km radius from their workplace) suggests the contribution of an additional source of exposure, especially on the NS group (Figure 5). BAS are also important for allergenic responses, which can be associated with increased air pollution originated from urban traffic (135–137); higher BAS were observed in firefighters from Bragança, Mirandela and Vinhais that acknowledged exposure to smoke (5–10 km radius from their workplace; Supplementary Figure S2). As mentioned previously, within the district and, in comparison to Carrazeda de Ansiães, Bragança is a bigger city with higher road traffic. Similarly, Mirandela has an aeroclub, an industrial zone, and a highway, two national and one municipal roads within 2–4 km of the Mirandela fire station, which contributes to air pollution in the city (138). Regarding Vinhais, there is only a national road, which could mean that there might be other sources of health relevant agents in this fire station in comparison to Carrazeda de Ansiães and thus further studies are needed. Moreover, there seems to be a cumulative effect of smoking habits and other environmental exposures at the fire stations that might be related to the increase of blood BAS in firefighters.

Besides relevant exposure to exogenous agents, endogenous characteristics and lifestyle can contribute to altered immunological parameters (67, 71, 139). Less active firefighters presented lower LYM than those that are more active (Supplementary Table S3). Recurrent exercise has been associated with a decrease in LYM due to the migration of these differentiated cells out of blood during the recovery phase (140). Moreover, more hours of work were associated with lower NEU and higher LYM among firefighters (Supplementary Table S3). The NEU are the first line of defense against pathogens as part of the innate immune response, while LYM will appear later on as part of the adaptative immune response previously triggered by NEU activity (141). Stress conditions can upregulate adaptative immunity while recruiting innate response cell out of the blood stream (142). An altered distribution of leucocyte subsets was observed in students who endured acute academic stress (143). Furthermore, one of the causes of chronic stress is sleep deprivation, which can also alter the body’s defense mechanisms (142). Night work-shifts were also associated with increased LYM in health workers (144). Consequently, firefighters who spend more hours at work can present cumulative stress and sleep pattern disruption/sleep deprivation due to night-shifts (145). Therefore, stress/sleep disruption could be a possible trigger for blood cell count alterations, yet to the best of the authors’ knowledge, there are no studies that have explored the impact of working hours on hematological parameters in this occupational context. On the other hand, regarding firefighters’ function, most drivers were non-smokers (56%) and physically active (69%), which could be one of the reasons why they displayed a lower percentage of MON and ALY. However, future studies need to better characterize the impact of job function on firefighters’ health.

Concerning the erythrocyte and platelets in firefighters blood (Table 2), the available literature has reported similar mean values before exposure to fire combat training for South Korean fire service instructors, i.e., 4.9 × 10^12^/L for RBC, 9.5–9.6 mmol/L for HGB, 45.2–46.3 ± 1.7% for HCT, 92.9–94.5 fL for MCV, 1.94–1.95 fmol for MCH, and 2.42 × 10^11^/L for PLT (146), and in UK firefighters, i.e., 2.09 × 10^11^/L for PLT (34).

Tobacco consumption can stimulate the production of red blood cells. Smoking is associated with the inhalation of carbon monoxide (CO). Once hemoglobin adsorbs CO, an irreversible reaction occurs, and erythrocytes can no longer transport oxygen. Thus, the renewal of these cells is stimulated and a consequent rise in their number can be observed in smokers (126) and corroborate the significant higher HCT, borderline non-significant higher RBC and HBG that was found in this study (Table 2). On the other hand, a reduction in the percentage of RDW by itself among smokers with less years of service (Figure 3A) does not have clinical significance. It must be crossed with other hemogram parameters, and depending on the results, the RDW could be used for differential anemia diagnosis (147). Smoking has been associated with higher RDW (147), yet, it is possible that smoking firefighters with a higher number of years of service (moderate/strong intercorrelation of smoking duration with years of service, and number of smoked cigarettes/day; Figure 3A), might produce more macrocytic RBCs, thus their size is more similar, and consequently, the subjects present a lower RDW with the increased number of years of service.

Trace smoke from fossil fuel vehicles exhaust (e.g., in closed garages), PPE storage rooms, and common areas with tobacco smoke may contain CO, which can irreversibly react with hemoglobin, thus a possible reason for the slight decrease in MCHC found for firefighters who acknowledged exposure to PM and/or gaseous pollutants during their work-shift (Supplementary Table S3). Also subjects who self-reported exposure to solvents had lower HGB and higher PCT, which was also observed for firefighters who were drivers (Supplementary Table S3). Similar findings have been observed in Pakistani auto-repair workers exposed to aromatic solvents (148). In fact, at fire stations, the lack of engine exhaust hoods in the garages can contribute to higher air levels of benzene, ethylbenzene, toluene, and xylene (149). Since some of the characterized Portuguese fire stations have a direct connection between the garage and the common areas inside the building, it is possible that the differences in HGB and PLTs are related to exposure to emissions from the heavy motor vehicles that can also cross-contaminate the air inside the headquarters. On the other hand, decreased indoor air quality can have hematopoietic effects (134). The finding of higher MCV and MCH in NS firefighters who self-report exposure to smoke within a 5–10 km radius from work (Figures 5C,D) suggests a possible association between decreased air quality due to smoke at the fire station surroundings and its contribution to larger red blood cells with a higher content of hemoglobin protein in firefighters.

Acute, strenuous exercise provided by a low frequency activity can lead to platelet activation, while regular physical activity can decrease/prevent platelet activation and favorably modulate platelet function (130, 150, 151). This could help to explain the findings of lower PCT and PLT among firefighters who exercise weekly (Supplementary Table S3), corroborating the positive correlations that were found between BMI and PLT (S group), MPV (NS group), and PCT (all firefighters) (Figure 3). A higher BMI has been related with platelet activation, thus, an associated inflammation and increased thrombosis risk (152–154). Therefore, these findings indicate that the characterized firefighters could benefit from including regular exercise in their routine to reduce cardiovascular disease risk. Accordingly, Durand et al. (155) reported that vigorous physical activity can reduce cardiometabolic profile parameters such as blood cholesterol-HDL ratio, triglycerides, glucose, and HDL increments in USA firefighters. Moreover, a recent study performed in male BALB/c mice observed that exercise can positively impact inflammatory cytokines and modulate gene expression related with REDOX imbalance induced by PAHs exposure (156).

### 4.4 Correlations between biomarkers of exposure and health (bio)markers

The lack of association between individual or ∑OHPAHs and higher blood pressure contrast with recent studies that reported significant correlations for in USA, Chinese, and South Korean petrochemical industry workers, coke oven workers, and chimney sweeps (12, 157). However, in this study, firefighters were not exposed to fire emissions for at least 1 week before sample collection, thus, lower metabolites concentrations were expected. Despite that, it is possible that the workers mentioned have far greater exposures at their workplace than firefighters have at their headquarters. A recent meta-analysis gathered information from available studies on the general population, which showed an overall meta-association between individual OHPAHs and blood pressure. However, after trim and fill analysis, no association was found (157). Also, subgroup analysis revealed an important contribution of USA and Asian ethnicity to the previous positive relationship found between urinary OHPAHs and blood pressure (157). Moreover, the high percentage of smokers and overweight subjects may pose some limitations to the obtained results in firefighters because a higher BMI, lower physical activity, and tobacco smoking are known risk factors for hypertension development.

Recent studies have associated PAHs exposure with inflammatory processes (21, 157–160). The obtained correlations found for 2-OHFlu (with MON and BAS) and 1-OHPyr (MON, BAS, ALY, and MCV) suggest a possible contribution from cigarette smoke exposure. Smoking is a recognized risk factor for cardiovascular disease development, whose mechanisms are mainly related with inflammation through increased leucocyte count and mutagenic alterations (127, 129, 151, 159, 161, 162). The negative correlations of NEU with 2-OHFlu and EOS with 1-OHPyr only in the NS group suggest that (without tobacco consumption as a variable for increased inflammation) there can be a possible smoking-independent negative impact of the two respective PAHs (fluorene and pyrene) on the first line of immune defense (NEU) and allergenic pathways (EOS). A recent study has identified a relationship between years of service as a firefighter and increased leucocyte epigenetic age (162). Since there were significantly older firefighters in the NS than in the S group and a strong correlation between years of service and age, it is plausible that these results (NEU and EOS) can also be age-related in the NS group. On the other hand, being a smoker, along with occupational exposure to air pollutants synergistically increases individual susceptibility to higher cardio-respiratory health effects, this is mainly through overstimulation of oxidative stress and inflammatory processes in the body caused by chronic exposure to toxic compounds, including PAHs (27, 72, 86, 161).

Inflammation has been associated with increased values of blood immune cell counts (163, 164), and a recent review reported that activated macrophages (matured MON) and regulatory T cells can have a protective role against hypertension (165). The inverse correlations between blood pressure parameters and MON, LIC, and RDW values could reflect a latent immune response state in firefighters with increased blood pressure. Thus, suggesting a decreased presence of MON (migrated and transformed into macrophages), the cells with the biggest size, while other WBCs have similar sizes, therefore culminating in a reduced LIC and RDW. Elevated heart rate itself has been independently associated with augmented immune cell count and inflammation biomarkers (166–168), corroborating the correlations found in this study, i.e., cardiac frequency with MON (NS group) and BAS (S group). Once more, there seems to be a cumulative contribution of smoking status to these associations. Tobacco consumption has been previously associated with hypertension, increased cardiac frequency, and immune cell counts (118, 119, 129, 169). All routes, i.e., respiratory, dermal, and gastrointestinal, can be responsible for cardiovascular effects of PAHs exposure (14). However, inhalation is the major concern among firefighters at fire combat scenarios. Nevertheless, the other routes cannot be overlooked, especially because dermal contact has been identified as a potential source of PAHs in firefighting occupational context and the gastronomic culture of these Portuguese firefighters includes smoked/processed meat, expanding their exposome.

## 5 Conclusion

This study provided evidence of high exposure to PAHs in Portuguese firefighters without exposure to fire combat activities during the last week, by assessing the urinary levels of six OHPAHs while evaluating health status based on blood pressure, cardiac frequency, and hemogram parameters. Therefore, it contributes to an occupational exposure-based input for PAHs biomonitoring and supports ongoing efforts to track and mitigate PAHs exposure to reduce inherent health risks for the European population. Overall creatinine-corrected ΣOHPAHs levels varied from 1.20 × 10^–1^ to 78.20 μmol/mol creatinine, which were significantly higher in smokers. Subjects who acknowledged having exposure to PM and/or gaseous pollutants during their work-shift at the fire stations presented significantly higher urinary 1-OHPyr levels, which were still below the occupational recommended level of 2.5 μg/L proposed by ACGIH. This study presented 2 to 10-fold higher ΣOHPAHs compared to the available data concerning firefighters without exposure to fire emissions. The baseline ΣOHPAHs concentrations were 2–23 times higher than in general populations from Europe and the USA, while remaining 2-fold lower than in Chinese. However, all individual metabolite levels, except for 1-OHNaph+1-OHAce, were below those reported for USA, Italian, German, and Portuguese populations. Thus, further investigation of PAHs exposure in firefighters during regular occupational activities at fire stations is warranted. The urinary biomarkers 2-OHFlu and 1-OHPyr have the potential to discriminate tobacco consumption. Smoking firefighters were predominantly hypertensive, which was attributed to smoking duration, age, and years of service of the subjects. Firefighters in the permanent intervention team presented higher systolic pressure than those who had other functions. On the other hand, despite hemogram parameters being within the recommended values, exercising regularly (weekly) and spending less than 8 h per day at the fire station seemed to have a protective role in firefighters’ health, whereas smoking was associated with higher BAS and HCT. A non-significant association was found between individual or ΣOHPAHs and blood pressure. On the other hand, blood pressure was inversely correlated with MON, LIC, and RDW. Individual PAH metabolites were positively correlated with differentiated leucocyte counts, but smoking could have been a possible contributor to these associations.

Future studies should aim to (i) enroll a greater number of females, if feasible, to more accurately characterize the exposure and health of women firefighters statistically; (ii) establish cross-contamination sources of PAHs and other pollutants at fire stations; (iii) establish follow-up studies to detect possible alterations in the blood pressure and hemogram of firefighters; (iv) explore the differences in lifestyle between firefighters from other countries; and (v) apply interventive measures to promote a healthier lifestyle among firefighters. Also, the use of biomarkers of relevant toxic mechanisms, such as oxidative stress, DNA damage, lung injury, etc., would be highly valuable to comprehensively characterize this population.

## Data availability statement

The original contributions presented in the study are included in the article/Supplementary material, further inquiries can be directed to the corresponding author.

## Ethics statement

The studies involving humans were approved by Accredited Ethics Committee of the University of Porto, Portugal, Report Nr. 92/CEUP/2020, under the project BioFirEx project (PCIF/SSO/0017/2018): “A panel of (bio)markers for the surveillance of firefighter’s health and safety.” The studies were conducted in accordance with the local legislation and institutional requirements. The participants provided their written informed consent to participate in this study.

## Author contributions

BB: Conceptualization, Data curation, Formal analysis, Methodology, Writing – original draft. AP: Conceptualization, Data curation, Formal analysis, Methodology, Writing – original draft. MO: Conceptualization, Methodology, Supervision, Validation, Writing – review & editing, Funding acquisition. SA: Conceptualization, Data curation, Methodology, Validation, Writing – review & editing. FE: Conceptualization, Data curation, Methodology, Validation, Writing – review & editing. AF: Investigation, Methodology, Supervision, Writing – review & editing. JV: Investigation, Methodology, Supervision, Writing – review & editing. KS: Investigation, Methodology, Supervision, Writing – review & editing. SC: Funding acquisition, Project administration, Supervision, Writing – review & editing. JT: Funding acquisition, Project administration, Supervision, Writing – review & editing. SM: Conceptualization, Funding acquisition, Methodology, Project administration, Supervision, Validation, Writing – review & editing.
