# Supplementary material for: Baseline data and associations between urinary biomarkers of polycyclic aromatic hydrocarbons, blood pressure, hemogram, and lifestyle among wildland firefighters
Source: Front Public Health. 2024 Mar 6;12:1338435. doi: 10.3389/fpubh.2024.1338435 (PMC10950961; doi:10.3389/fpubh.2024.1338435)
Supplement: Supplementary file 1 [file Data_Sheet_1.docx]

Supplementary Material

Baseline data and associations between urinary biomarkers of polycyclic aromatic hydrocarbons, blood pressure, hemogram, and lifestyle among wildland firefighters

Bela Barros^1^; Ana Margarida Paiva^1^, Marta Oliveira^1^, Sara Alves^2^, Filipa Esteves^3,4,5^, Adília Fernandes^2^, Josiana Vaz^6,7^, Klara Slezakova^8^, Solange Costa^3,5^, João Paulo Teixeira^3,5^, Simone Morais^1*^

^1^ REQUIMTE/LAQV, Instituto Superior de Engenharia do Porto, Instituto Politécnico do Porto, Porto, Portugal

^2^ Instituto Politécnico de Bragança, UICISA: E, Unidade de Investigação em Ciências da Saúde: Enfermagem, Instituto Politécnico de Bragança Campus de Santa Apolónia, Bragança, Portugal

^3^ Environmental Health Department, National Institute of Health Dr. Ricardo Jorge, Porto, Portugal

^4^ Department of Public Health and Forensic Sciences, and Medical School, Faculty of Medicine, University of Porto, Porto, Portugal

^5^ EPIUnit - Instituto de Saúde Pública da Universidade do Porto, Porto, Portugal

^6^ CIMO, Instituto Politécnico de Bragança, Bragança, Centro de Investigação de Montanha Campus Santa Apolónia, Bragança, Portugal

^7^ SusTEC, Instituto Politécnico de Bragança, Bragança, Sustec - Associate Laboratory for Sustainability and Technology in Inland Regions - Campus Santa Apolónia, Bragança, Portugal

^8^ LEPABE-ALiCE, Departamento de Engenharia Química, Faculdade de Engenharia, Rua Dr. Roberto Frias, Porto, Portugal

*** Correspondence:**Simone Morais
sbm@isep.ipp.pt

# Supplementary Materials and Methods

## Study population (additional information)

Alto Trás os Montes of the Northern region of Portugal is one of the region most affected by large and intense wildfires due to its characteristic mountain areas, increasing drought, and topographic conditions (1,2). In fact, from 2001-2021, Bragança district lost almost 49% of its tree cover due to fires (3). This Portuguese district has an area of approximately 6600 km^2^ divided into 12 municipalities: Bragança, Mirandela, Macedo de Cavaleiros, Mogadouro, Vinhais), Torre de Moncorvo, Miranda do Douro, Vila Flor, Carrazeda de Ansiães, Alfândega da Fé, Vimioso, and Freixo de Espada à Cinta. Bragança municipalities are mainly characterized by their traditional province side at higher altitudes (218-763 m above sea level), and low population density (inhabitants/km²), i.e., Mirandela (32.5) > Bragança (29.5) > Vila Flor (22.8) > Macedo de Cavaleiros (20.4) Carrazeda de Ansiães (19.7) > Alfândega da Fé (13.4) > Miranda do Douro (13.3) > Freixo de Espada à Cinta (13.2) > Torre de Moncorvo (12.8) > Vinhais (11.2) > Mogadouro (10.9) > Vimioso (8.6); (4). These characteristics contrast with what is found in the main urban coastline Portuguese municipalities, i.e., Lisbon (area: 85 km^2^; 5455.2 inhabitants/km²; altitude: 4 m) and Porto (area: 41 km^2^ and 5596.3 inhabitants/km²; altitude: 83 m).

# Supplementary Tables

**Supplementary Table S1.** Additional information of the characterized Portuguese* firefighters.

|  | **Non-Smoker (*n*=76)** | **Smoker (*n*=59)** | **Total (*n*=135)** |
| --- | --- | --- | --- |
| **Characteristic** | **%** (unless indicated otherwise) | **%** (unless indicated otherwise) | **%** (unless indicated otherwise) |
| Firefighter category |  |  |  |
| Chief | 4.6 | 3.8 | 4.3 |
| Subchief | 20.0 | 3.8 | 12.8 |
| 1^st^ grade firefighter | 10.8 | 9.6 | 10.3 |
| 2^nd^ grade firefighter | 18.5 | 19.2 | 18.8 |
| 3^rd^ grade firefighter | 44.6 | 59.6 | 51.3 |
| Other | 1.5 | 4.0 | 2.5 |
| Fire station |  |  |  |
| Bragança | 5.3 | 10.2 | 7.4 |
| Vinhais | 10.5 | 16.9 | 13.3 |
| Alfândega da Fé | 10.5 | 8.5 | 9.6 |
| Mogadouro | 9.2 | 8.5 | 8.9 |
| Mirandela | 6.6 | 13.6 | 9.6 |
| Torra de Dona Chama | 5.3 | 5.0 | 5.2 |
| Freixo de Espada à Cinta | 3.9 | 5.0 | 4.4 |
| Macedo de Cavaleiros | 3.9 | 11.9 | 7.4 |
| Izeda | 3.9 | 1.7 | 3.0 |
| Vimioso | 19.8 | 6.8 | 14.1 |
| Miranda do Douro | 5.3 | 1.7 | 3.7 |
| Vila Flor | 9.2 | 3.4 | 6.7 |
| Carrazeda de Ansiães | 6.6 | 6.8 | 6.7 |
| Firefighter function (yes) |  |  |  |
| Administrative board | 1.4 | 1.9 | 1.6 |
| Commander | 8.5 | 5.8 | 7.3 |
| Rescue | 2.8 | 7.7 | 4.9 |
| Diver | 4.2 | 5.8 | 4.9 |
| Paramedic | 28.2 | 34.6 | 30.9 |
| Driver | 28.2 | 30.8 | 29.3 |
| Telephone operator | 14.1 | 7.7 | 11.4 |
| Permanent Intervention Team | 35.2 | 34.6 | 35.0 |
| Stock management | 1.4 | m.d. | 0.8 |
| Other | 18.3 | 30.8 | 23.6 |
| Total number of functions at the fire station, mean ±SD (min.-max.)* | 1.42 ± 0.82 (1-5) | 1.60 ± 1.11 (1-6) | 1.50 ± 0.95 (1-6) |
| Number of hours spent at the fire station per day |  |  |  |
| < 8 h | 15.7 | 16.1 | 15.8 |
| 8-9h | 48.6 | 33.9 | 42.1 |
| ≥ 10h | 35.7 | 50.0 | 42.1 |
| Exposure during work-shift on a daily and/or weekly basis (yes) |  |  |  |
| Gaseous pollutants, particulate matter | 88.2 | 86.0 | 87.2 |
| Solvents | 29.9 | 26.9 | 28.6 |
| Living near (~500 m) - (yes) |  |  |  |
| Factory | 1.4 | 5.2 | 3.1 |
| Landfill/trash storage | m.d. | m.d. | m.d. |
| Industrial area | 5.9 | 5.5 | 5.7 |
| Living near (~200 m) farming area in which pesticides are used (yes) | 31.4 | 22.4 | 27.3 |
| Physical activity |  |  |  |
| No | 18.6 | 27.6 | 22.6 |
| Sometimes/year | 21.4 | 41.4 | 30.5 |
| Weekly | 47.1 | 27.6 | 38.3 |
| Daily | 12.9 | 3.4 | 8.6 |

* Independent-samples Mann-Whitney U test, p= 0.452; max.: maximum; min.: minimum; m.d.: missing data and the remaining individuals answered "No"; n.a.: not applicable; SD: Standard Deviation.

Supplementary Table S2. Background median (minimum-maximum) unadjusted levels of urinary biomarkers of PAHs exposure in Portuguese firefighters (data expressed as µg/L of urine).

|  | **Non-Smoker** |  | **Smoker** |  | ***p* value*** | **Total** |
| --- | --- | --- | --- | --- | --- | --- |
| **1-OHNaph+1-OHAce** | 31.42 (1.08 ×10^-1^- 430.13) | | 112.66 (6.49- 620.08) | | <0.001 | 63.90 (1.08 ×10^-1^- 620.08) |
| **2-OHFlu** | 0.08 (1.70 ×10^-3^- 5.20) | | 0.61 (1.70 ×10^-3^- 4.31) | | <0.001 | 0.15 (1.70 ×10^-3^- 5.20) |
| **1-OHPhe** | 0.13 (1.50 ×10^-3^- 1.28) | | 0.22 (1.68 ×10^-2^- 1.08) | | 0.003 | 0.15 (1.50 ×10^-3^ - 1.28) |
| **1-OHPyr** | 0.05 (6.00 ×10^-4^- 1.09) | | 0.12 (6.10 ×10^-3^-1.63) | | <0.001 | 0.07 (6.00 ×10^-4^- 1.63) |
| **Σ OHPAHs** | 33.60 (2.75 ×10^-1^- 430.24) | | 115.20 (1.21 ×10^1^- 621.14) | | <0.001 | 64.21 (2.75 ×10^-1^- 621.14) |

* Independent-samples Mann-Whitney U test, significance set at p<0.05.
The urinary metabolite of benzo(a)pyrene (3-hydroxybenzo(a)pyrene) was always below its detection limit.

Supplementary Table S3. Significant differences found between categorical variables from the applied questionnaire and (bio)markers evaluated in Portuguese firefighters. Independent-samples Mann-Whitney U test (unless indicated otherwise).

| **(Bio)marker** | **Median Ratio** |  |
| --- | --- | --- |
|  | **(yes/no)** | ***p* value** |
| **Exposure to smoke, gaseous pollutants, particulate matter during firefighter activities (yes *versus* no)** |  |  |
| 1-OHNaph+1-OHAce (µmol/mol creatinine) | 3.61 | 0.005 |
| Σ OHPAHs (µmol/mol creatinine) | 3.52 | 0.006 |
| MCHC (mmol/L) | 0.99 | 0.011 |
| **Exposure to solvents during firefighter activities (yes *versus* no)** |  |  |
| HGB (mmol/L) | 0.96 | 0.009 |
| PCT (%)* | 1.10 | 0.027 |
| **Physical activity (sometimes/year *versus* no)** |  |  |
| PLT (×10^11^/L)* | 1.15 | 0.019 |
| PDW (%)* | 0.91 | 0.005 |
| **Physical activity (weekly *versus* sometimes/year)** |  |  |
| LYM (×10^9^/L)* | 0.84 | 0.006 |
| PLT (×10^11^/L)* | 0.87 | 0.005 |
| PCT (%)* | 0.88 | 0.006 |
| **Number of hours spent at the fire station per day (≥10 *versus* 8-9h)** |  |  |
| NEU (%) | 0.93 | 0.038 |
| LYM (%)* | 1.11 | 0.022 |
| **Driver (yes *versus* no)** |  |  |
| MON (%) | 0.93 | 0.032 |
| ALY (%) | 0.90 | 0.021 |
| HGB (mmol/L) | 0.93 | <0.001 |
| HCT (%)* | 0.95 | 0.005 |
| PCT (%)* | 1.15 | 0.021 |
| **Permanent intervention team (yes *versus* no)** |  |  |
| Systolic pressure (mm Hg) | 1.05 | 0.036 |

*Independent samples *t* test was used, and the ratio of means is presented. ALY: Atypical lymphocytes; MON: Monocytes; HCT: Hematocrit; 1-OHNaph+1-OHAce: 1-hydroxynaphthalene+1-hydroxyacenaphtene; HGB: Hemoglobin; LYM: Lymphocytes; MCHC: Mean corpuscular hemoglobin concentration; NEU: Neutrophils; PCT: plateletcrit; PDW: Platelet distribution width; PLT: Platelet count; Σ OHPAHs: Sum of PAHs

# Supplementary Figures


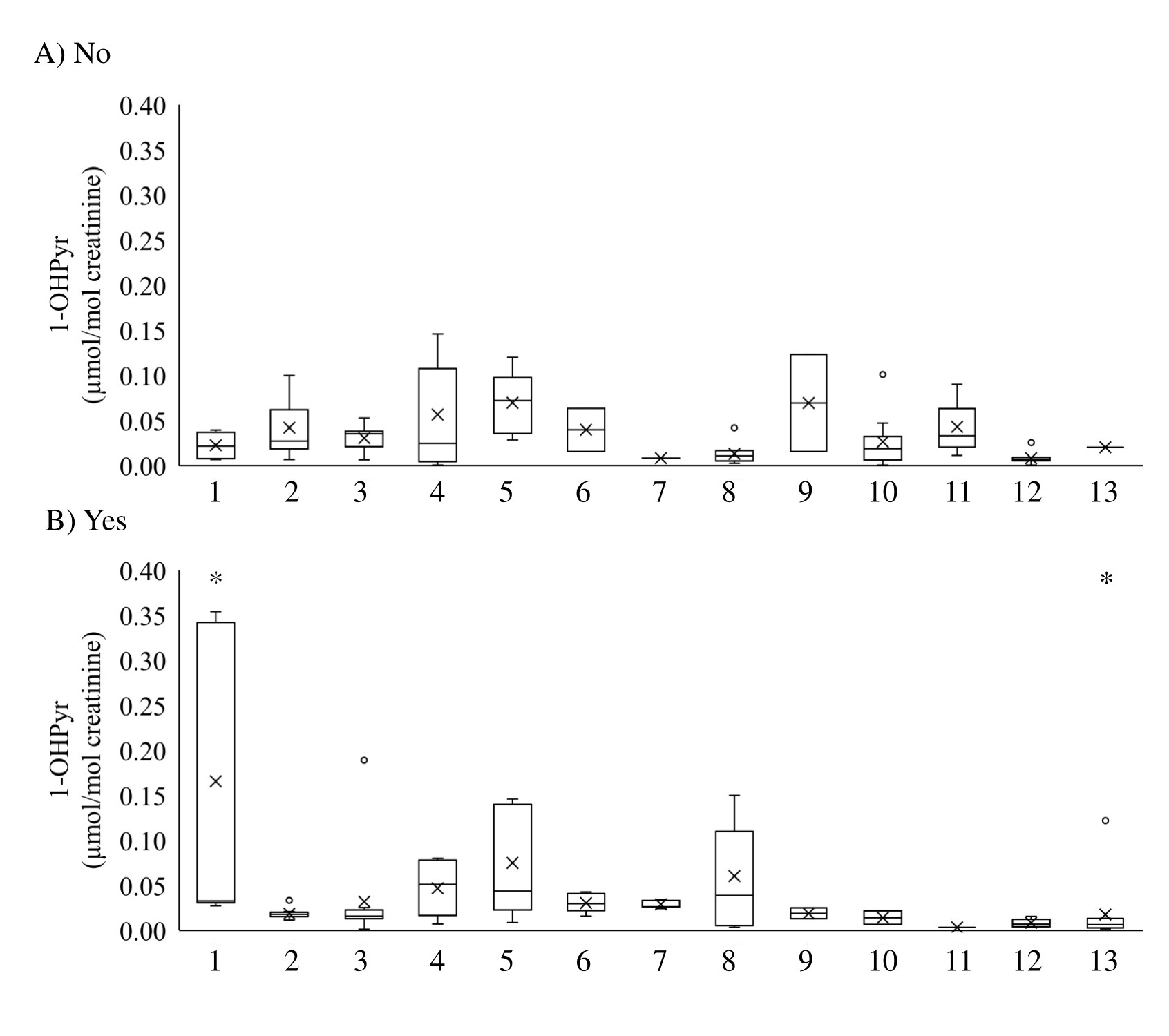


**Supplementary Figure S1.** Creatine adjusted 1-hydroxypyrene (1-OHPyr, µmol/mol creatinine) urinary concentrations in firefighters according with their answer to having additional exposure to smoke in a 5-10 km radius from their work (fire station; A: “No”; B: “Yes”). 1: Bragança; 2: Vinhais; 3: Alfândega da Fé; 4: Mogadouro; 5: Mirandela; 6: Torre de Dona Chama; 7: Freixo de Espada à Cinta; 8: Macedo de Cavaleiros; 9: Izeda; 10: Vimioso; 11: Miranda do Douro; 12: Vila Flor; 13: Carrazeda de Ansiães. *: Significant differences between Bragança and Carrazeda de Ansiães (p=0.034). Statistical significance set at p<0.05 using the independent-samples Kruskal-Wallis test.


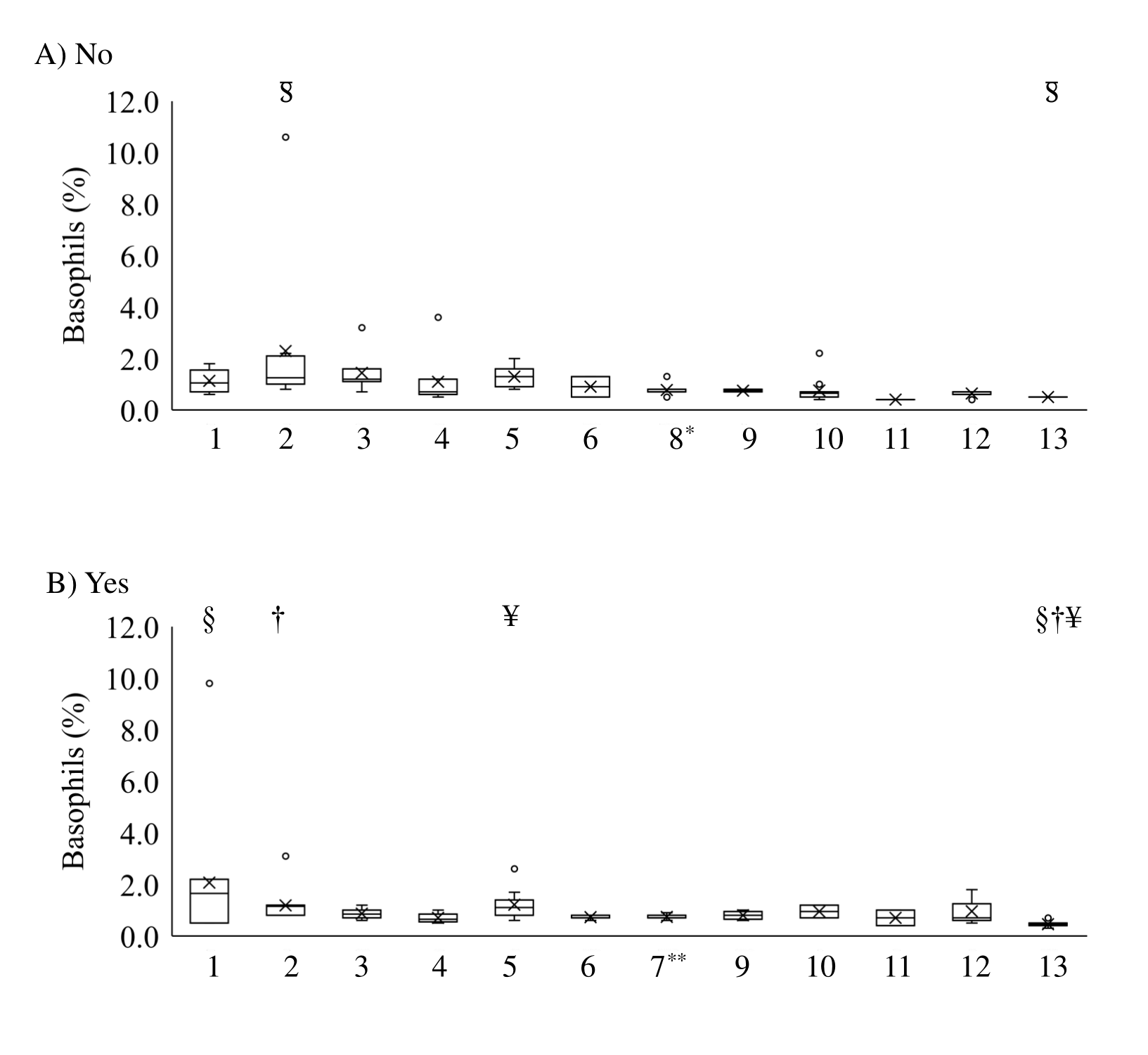


**Supplementary Figure S2.** Percentage of blood basophils among firefighters according with their answer to acknowledged exposure to smoke in a 5-10 km radius from work (fire station; A: “No”; B: “Yes”). 1: Bragança; 2: Vinhais; 3: Alfândega da Fé; 4: Mogadouro; 5: Mirandela; 6: Torre de Dona Chama; 7: Freixo de Espada à Cinta; 8: Macedo de Cavaleiros; 9: Izeda; 10: Vimioso; 11: Miranda do Douro; 12: Vila Flor; 13: Carrazeda de Ansiães). Significant differences between: ꟑ: Vinhais e Vimioso (*p*=0.009); §: Bragança and Carrazeda de Ansiães (*p*=0.038); † Vinhais and Carrazeda de Ansiães (*p*=0.004); ¥: Mirandela and Carrazeda de Ansiães (*p*=0.019). *: Note that all firefighters with hemogram analysis from Macedo de Cavaleiros answered “No”; **: Note that all firefighters with hemogram analysis from Freixo de Espada à Cinta answered “Yes”. Statistical significance set at *p*<0.05 using the independent-samples Kruskal-Wallis test.

# References

1. Cavalli A. Áreas ardidas e risco de erosão potencial em zonas de montanha do NE Portugal. [Mestrado com dupla diplomação com a Universidade Tecnológica Federal do Paraná]. Bragança: Instituto Politécnico de Bragança (2017).

2. ICNF (2022). 8.^o^ RELATÓRIO PROVISÓRIO DE INCÊNDIOS RURAIS DE 2022. *Divisão de Gestão do Programa de Fogos Rurais Instituto Da Conservação Da Natureza E Das Florestas.* Available at: https://www.icnf.pt/api/file/doc/4e8a66514175d0f7 (2022)

3. Tyukavina A, Potapov P, Hansen MC, Pickens AH, Stehman S V., Turubanova S, Parker D, Zalles V, Lima A, Kommareddy I, et al. Global Trends of Forest Loss Due to Fire From 2001 to 2019. *Fron. Remote Sens.* (2022) 3. doi: 10.3389/frsen.2022.825190

4. INE PORDATA (2022). CENSOS 2021 por concelho e regiões: Evolução 1960-2021. *INE, PORDATA Estatísticas Sobre Portugal e a Europa Fundação Manuel dos Santos*. Available at: https://www.pordata.pt/censos/quadro-resumo-municipios-e-regioes/braganca-446
